# Bridging the Evidence–Practice Gap in Early Burn Injury Care: A Comprehensive Evidence Synthesis of Global Guidelines, Consensus, and Systematic Reviews for Resource-Limited Settings

**DOI:** 10.3390/ebj7020034

**Published:** 2026-06-10

**Authors:** Hongyu Tang, Shenjing Yu, Rui Zhang, Zheng Zhu, Li Gui

**Affiliations:** 1 School of Nursing, Naval Medical University, Shanghai 200433, China; 15211170016@fudan.edu.cn (H.T.); emily19991027@163.com (S.Y.); 2 Department of Military Health Management, Naval Medical University, Shanghai 200433, China; zhangzz_2024@163.com; 3 School of Nursing, Fudan University, Shanghai 200032, China

**Keywords:** evidence-based, burn injury, emergency care, summary report, nursing

## Abstract

**Highlights:**

**What are the main findings?**

- 77 evidence-based recommendations across 13 domains establish an integrated burn emergency care framework.
- Evidence reveals a protocol-resource disconnect, where underrepresented ABC and system-level enablers critically determine acute outcomes

**What are the implications of the main findings?**

- Early burn care is system-based, requiring adaptive scene control and resource-calibrated interventions, not rigidity.
- ABC saves lives, while suboptimal interventions sustain life until transfer, yet require greater resource capacity.

**Abstract:**

Background: Early management of adult burn injuries in resource-constrained environments—such as battlefields and primary care facilities—remains hindered by the absence of standardized, evidence-based protocols. This study aimed to systematically synthesize existing evidence and develop an integrated framework of actionable recommendations to optimize prehospital and early emergency care. Methods: A comprehensive evidence synthesis was conducted across 14 international and domestic bibliographic databases and authoritative repositories. Eligible sources included clinical practice guidelines, expert consensus statements, evidence summaries, and systematic reviews. Literature quality was appraised using validated instruments, and best-practice recommendations were extracted and thematically synthesized across the continuum of early burn care. Results: Fifty-nine high-quality studies yielded 77 recommendations across 13 domains, spanning from scene safety and burn process cessation through airway, breathing, and circulatory management to wound care, infection control, and transfer preparation. An integrated, context-adaptive framework was established to guide resource-calibrated interventions rather than rigid protocol adherence. Conclusions: These findings provide tiered guidance for frontline healthcare providers and inform the development of emergency care standards in resource-limited settings. Future research should prioritize field validation and contextual implementation to address barriers to evidence translation and enhance real-world applicability.

## 1. Introduction

Burn injuries represent one of the most devastating forms of trauma, causing extensive tissue damage and systemic complications through exposure to thermal, chemical, electrical, or radiation sources [1]. Globally, burns are a persistent public health burden, with an estimated 180,000 deaths annually and a disproportionate concentration in low- and middle-income countries (LMICs) [2,3,4]. According to the Global Burden of Disease 2019 study, approximately 9 million new burn cases occur each year, with mortality rates showing a worrisome upward trend in certain regions [5]. In addition to civilian contexts, burns remain a major cause of morbidity and mortality during armed conflicts [6]. Data from historical and contemporary military operations, including the current Russia–Ukraine conflict, consistently demonstrate the high incidence and severity of thermal injuries among combat casualties [7].

Improper first aid or delayed early burn management can directly increase complications and even mortality [8]. Evidence indicates that timely and standardized early management can significantly limit burn depth progression, reduce infection risk, and improve long-term prognosis [8]. However, in real-world primary care settings, early management of adult burn patients is often performed by general practitioners or community health workers, who lack specialized training and face constraints in equipment, medication, and access to specialized knowledge and evidence in burn treatment [9]. Meanwhile, primary care providers in resource-constrained settings often rely on empirical judgment or seek consultation from specialized burn physicians, lacking comprehensive and evidence-based guidance, which is plagued by issues such as low accuracy in burn injury assessment and the presence of hidden shock in burn cases [10]. This leads to wide variability in the quality of care and frequent reliance on empirical or outdated practices, potentially exacerbating injury severity and delaying recovery [11].

Despite the availability of numerous international guidelines and expert consensus documents [12], several systemic barriers hinder the effective translation of evidence into early clinical practice. First, recommendations for the early management phase are fragmented across different guidelines and chapters, lacking consolidation for time-sensitive decision-making. Second, most guidelines are designed for tertiary burn centers with optimal infrastructure, rendering many recommendations impractical for low-resource environments. Finally, limited cross-context validation and inadequate dissemination strategies have perpetuated the evidence–practice gap, leaving primary health care providers without accessible, standardized, and actionable frameworks.

To address this unmet need, the present study systematically integrates high-quality evidence (obtained through rigorous evidence evaluation and tracing the evidence level from original studies) from global guidelines, expert consensus, and systematic reviews to develop a concise, practice-oriented evidence summary for early management of adult burn patients. By emphasizing applicability in resource-limited contexts, this synthesis aims to provide an operational foundation for standardized emergency care, enhance the consistency of frontline responses, and inform future policy and protocol development for adult burn injury management.

## 2. Materials and Methods

This study followed the methodological framework of the Joanna Briggs Institute (JBI) for evidence synthesis and reporting of evidence summaries. The process was conducted in accordance with the updated 2021 JBI Manual for Evidence Implementation, complemented by the 5S evidence pyramid model for hierarchical retrieval of pre-appraised evidence sources [13,14].

### 2.1. Formulation of Research Questions

Research questions were formulated using the PIPOST framework, defining the following components: Population (P): adult patients with burn injuries (mainly thermal, or mixed types); Intervention (I): emergency care or early care interventions of burn injuries; Professional (P): healthcare providers or emergency responders involved in early management; Outcome (O): indicators of early care effectiveness (e.g., survival, infection prevention, stability of basic vital signs prior to transportation); Setting (S): primary care facilities, prehospital settings, or battlefield prior to specialized burn center transfer; Type of evidence (T): guidelines, expert consensus, evidence summaries, and systematic reviews.

### 2.2. Data Sources and Search Strategy

A comprehensive and systematic search was conducted across 14 authoritative electronic databases and institutional websites from inception to March 2025. These included major clinical guideline repositories, such as the National Institute for Health and Care Excellence (NICE), Registered Nurses Association of Ontario (RNAO), Agency for Healthcare Research and Quality (AHRQ), and Chinese Yimaitong website; literature databases, such as UpToDate, Cochrane Library, JBI Evidence—based Database, PubMed, Embase, Web of Science, China Knowledge Network (CNKI), China Biomedical Literature Database (CBM); as well as academic society official websites, such as International Society for Burn Injury (ISBI), and American Burn Association (ABA). Search terms were developed using both controlled vocabulary (MeSH/Emtree) and free-text terms, incorporating Boolean logic and truncations to capture British and American spellings. An English database search using PubMed with the search strategy was as follows: (“burns” OR “burn injuries” OR “thermal injuries” OR “burn*” OR “burn injury” OR “burn wound*”) AND (“emergency treatment” OR “first aid” OR “emergency care” OR “emergency nursing” OR “early care” OR “pre-hospital care” OR “prehospital management” OR “emergency care” OR “emergency management” OR “acute care” OR “resource-limited” OR “low-resource” OR “austere environment”) AND (“practice guidelines” OR “guideline” OR “consensus” OR ”expert consensus” OR “systematic review” OR “meta-analysis” OR “evidence-based practice” OR “evidence synthesis” OR “evidence summary” OR “recommendation*”). Equivalent search strategies were adapted for Embase (Emtree terms), Web of Science, Cochrane Library (MeSH descriptor explosions), and major Chinese databases. All retrieved records were exported into EndNote X9 and deduplicated automatically and manually. Other new studies were identified through citation searching.

### 2.3. Inclusion and Exclusion Criteria

Inclusion criteria for this study were (1) research topics that focus on burn emergency care plans, encompassing burn care measures, procedures, and decisions; (2) study subjects were limited to adult burn patients or casualties with burn injuries; (3) study types were practice guidelines, systematic reviews, evidence summaries, and expert consensus; (4) English and Chinese studies.

Exclusion criteria were (1) duplicate inclusion or verbatim translation of studies; (2) interpretation of studies; (3) content only concerning burn rehabilitation treatment or training; (4) studies not available through various channels; (5) existing updated versions of literature.

### 2.4. Screening and Selection Process

**Figure 1:**
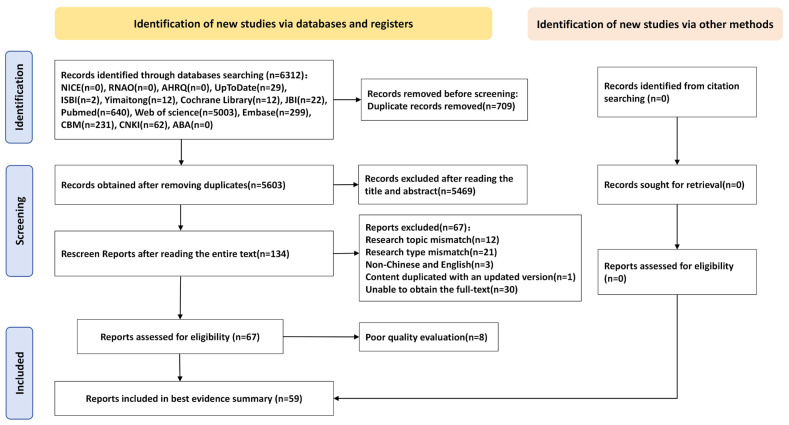
Flow chart of literature screening.

Two reviewers (HT and SY) independently screened all titles and abstracts. The full text of potentially relevant studies was retrieved and evaluated against eligibility criteria. Discrepancies were resolved through discussion or adjudication by a third senior reviewer (LG). The screening process followed the PRISMA 2020 flow (Figure 1).

### 2.5. Quality Appraisal

Quality appraisal was conducted independently by three reviewers (HT, SY, and RZ) trained at the Fudan University JBI Center. Guidelines were assessed using the AGREE II instrument, which includes 23 items across six domains: scope and purpose, stakeholder involvement, rigor of development, clarity, applicability, and editorial independence. Each item was scored from 1 to 7, and standardized domain percentages were computed to categorize quality as level A (strong recommendation, ≥60% in all domains), level B (recommended, ≥30% in at least three domains), or level C (not recommended, <30% in three or more domains). Systematic reviews were appraised using the 11-item JBI checklist for systematic reviews and research syntheses, while expert consensus was evaluated using the JBI Evidence-Based Healthcare Evaluation Criteria (2016 edition). Given the lack of a validated instrument for assessing evidence summaries, we traced each summary back to its primary studies and applied the appraisal tools recommended in the 2016 JBI Evidence-Based Healthcare Center standards [15]. Inter-rater reliability was calculated using the intraclass correlation coefficient (ICC), with ICC ≥ 0.75 interpreted as excellent agreement.

### 2.6. Data Extraction

Data extraction was carried out using a pre-piloted Excel form. Two reviewers (HT and SY) independently captured the authors, publication dates, titles, and topics of the included studies, along with the study types (guidelines, expert consensus, systematic reviews, or evidence summaries), evidence entry content, and evidence levels. Any discrepancies were discussed until consensus was achieved, with arbitration by a senior investigator (ZZ) when required.

### 2.7. Evidence Synthesis and Integration

Evidence synthesis followed a hierarchical inductive approach. Recommendations were first grouped by clinical domain, such as airway management, circulatory resuscitation, pain control, wound care, and infection prevention. Overlapping evidence was merged by content similarity, and conflicting findings were resolved by prioritizing recent, high-quality, and methodologically rigorous sources. Evidence was stratified into first-line essential measures and second-line context-dependent measures to reflect clinical feasibility under varying resource conditions. Two reviewers (HT and SY) independently conducted the merging process to minimize subjective bias, comparing wording, conceptual overlap, and underlying evidence sources. Each independently merged dataset was then cross-checked for consistency. When discrepancies occurred—such as divergent interpretations of overlapping statements or inconsistent classification across domains—they were discussed in consensus meetings moderated by a senior reviewer (LG). Disagreements that could not be resolved through discussion were referred to a third methodological expert for arbitration. All merging decisions, rationale for inclusion or exclusion, and any modification of wording were documented in an audit trail maintained in Microsoft Excel and archived in the project repository. All final recommendations were tabulated and narratively summarized following JBI standards.

### 2.8. Quality Assurance and Peer Validation

Quality assurance was ensured through a structured peer-review process. Reviewers were required to meet the following eligibility criteria: a minimum of 10 years of experience in burn medicine or specialized/critical care nursing; possession of a senior professional title or a postgraduate degree; and voluntary participation in the study. In accordance with the JBI recommendation system, evidence was categorized into two levels: A-level (strong recommendation) and B-level (weak recommendation) [15]. The research team first conducted a preliminary appraisal of the strength of each of the 77 aggregated evidence statements. Subsequently, an online external expert-consultation workshop was held, in which five senior burn or trauma emergency-care specialists independently reviewed the initial ratings. Discrepancies between expert assessments and the preliminary evaluations were addressed through group deliberation and resolved by adjudication from the team leader. Each evidence statement was examined for feasibility, clarity, and contextual relevance (especially the applicability of developed-world evidence to resource-limited contexts). Expert feedback was incorporated into the final synthesis through iterative consensus rounds.

## 3. Results

### 3.1. Study Selection and Characteristics

A total of 6312 records were retrieved through the database and manual searches. After removal of 709 duplicates, 5603 unique citations were screened by title and abstract. Of these, 104 full-text articles were reviewed, and 67 publications ultimately met the inclusion criteria. The included literature comprised 10 clinical practice guidelines [12,16,17,18,19,20,21,22,23,24], 26 expert consensus statements [25,26,27,28,29,30,31,32,33,34,35,36,37,38,39,40,41,42,43,44,45,46,47,48,49,50], 24 systematic reviews [51,52,53,54,55,56,57,58,59,60,61,62,63,64,65,66,67,68,69,70,71,72,73,74], and 7 evidence summaries [75,76,77,78,79,80,81]. The PRISMA 2020 flow diagram for study selection is shown in Figure 1. The primary research locations spanned 20 countries across five continents. China contributed the largest number of studies (*n* = 19) [25,26,27,29,30,31,32,36,43,45,46,54,65,68,75,76,78,79,80], followed by the United States (*n* = 12) [12,16,17,21,23,33,37,38,39,40,41,77], Australia (*n* = 9) [28,34,35,47,63,69,71,72,81], and the United Kingdom (*n* = 5) [19,20,44,50,67]. The remaining 21 studies were conducted across 16 other countries. The literature included both civilian and military burn-care contexts. Most studies (82.09%) were published after 2015, and 40.30% of these were published in the past five years. General characteristics of the included studies are shown in File S2.

### 3.2. Quality Appraisal of Included Evidence

Methodological appraisal demonstrated overall moderate-to-high quality among the included studies, though inter-source variability was evident. After literature screening, 10 guidelines were selected for quality assessment using the AGREE II instrument (Table 1). Among them, 9 were classified as a level B rating [12,16,17,18,19,21,22,23,24] and 1 as level C (indicating poor quality), thus being excluded. Ultimately, 9 guidelines were included for evidence item extraction. The strongest performing domains were “scope and purpose” and “clarity of presentation,” while “applicability” and “editorial independence” exhibited lower scores, reflecting ongoing challenges in operationalization and conflict-of-interest management. Interrater reliability across reviewers reached ICC = 0.844, indicating strong agreement.

Among the 26 expert consensus statements, 14 received “Yes” for all evaluation criteria, reflecting high methodological rigor [25,26,27,29,30,31,32,33,36,38,39,40,44,48]; ten were of moderate quality [28,34,35,37,41,43,45,46,47,50], mainly limited by insufficient reporting of evidence sources or the Delphi process, and two were excluded due to poor transparency and outdated evidence [42,49].

Twenty-four systematic reviews were appraised using the JBI 11-item checklist (Table 2). Six reviews were rated as high quality [51,54,58,62,65,66], thirteen as moderate quality [53,55,57,59,60,61,64,68,70,71,72,73,74], and five as low quality and excluded [52,55,56,63,67,69]. High-quality reviews consistently employed comprehensive search strategies and transparent data extraction processes.

The seven evidence summaries were all retained, having demonstrated adequate methodological reporting and clear linkage between evidence sources and recommendations [75,76,77,78,79,80,81].

### 3.3. Summary of Evidence and Practice Recommendation Results

**Figure 2:**
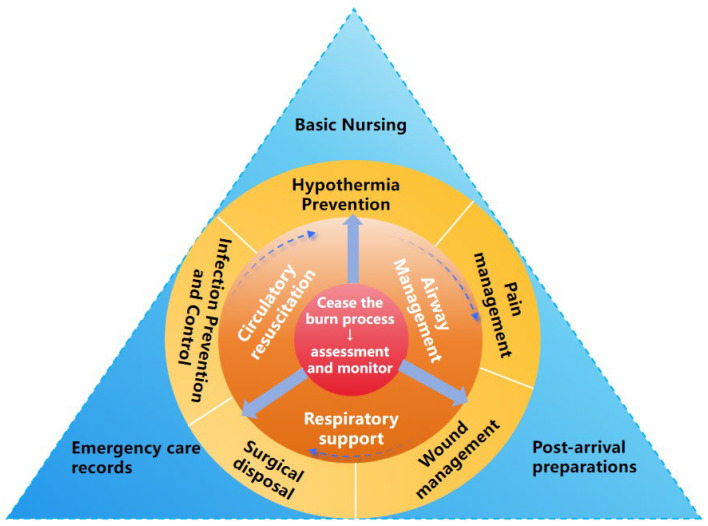
Framework of early management of adult burn patients. Note: The framework is arranged in concentric circles from the center outward to distinguish the priority of various measures for early management of adult burn patients. Different colors represent distinct levels: red denotes the core interventions; orange circles indicate the first level; yellow represents the second level; and blue signifies the third layer. Blue solid arrows illustrate the sequential progression of handling measures from inside to outside, while blue dashed arrows suggest that the ABC emergency response measures may be implemented in a cyclical manner without strictly fixed order.

Following critical appraisal, 59 articles met the inclusion criteria for evidence extraction and synthesis. From these studies, the research team derived 77 evidence statements, which were organized into 13 overarching themes representing key components of early management prior to specialized burn care. These themes encompassed cessation of the burn process, early assessment and monitoring, airway management, respiratory support, circulatory resuscitation, prevention of hypothermia, pain control, wound care, infection prevention, surgical management, nursing care, documentation, and patient transfer. The synthesized evidence is presented in Table 3. Based on methodological quality and contextual feasibility, all synthesized recommendations were stratified into one core and three hierarchical levels. The core interventions were the immediate cessation of the burn process coupled with prompt assessment and monitoring, an unequivocally non-negotiable emergency. The first-tier comprised essential, time-sensitive interventions universally applicable across clinical contexts, including airway management, respiratory support, and circulatory resuscitation. The second level encompassed context-dependent interventions, such as hypothermia prevention, pain management, wound protection, surgical decompression, and infection control—whose applicability depends on available infrastructure and personnel expertise. The third layer encompassed essential interventions or procedures, including basic nursing care, emergency care documentation, and post-arrival and transfer preparations. The hierarchical relationship and priority order among the topics formed the basic framework of early management of adult burn patients, shown in Figure 2.

## 4. Discussion

### 4.1. Safety and On-Site Control as Prerequisites for Effective Burn Injury Management

Ensuring scene safety and establishing on-site control are essential prerequisites for the effective management of burn victims. Continuous energy transfer—whether from residual heat, open flames, chemicals, or electrical currents—can significantly exacerbate tissue destruction [83,84]. Thus, the immediate removal of the injured individual from thermal or hazardous source represents the critical first step in early burn care and forms the foundation for all subsequent rescue procedures. This phase requires seamless coordination between fire-rescue personnel and trained first-aid responders. The evidence supporting these initial measures is substantial and largely consistent, typically implemented by primary health care providers such as pre-hospital emergency ambulance personnel, trained in basic life support rather than specialized burn care, and requiring relatively limited medical resources [85]. Given the inherent unpredictability of on-site conditions, responders must first ensure environmental safety to protect both themselves and the victim. Rescue preparations must take into account the substantial differences between field conditions and controlled hospital settings, underscoring the need for responders to demonstrate situational adaptability and strong self-protection awareness [86]. Special caution is required during the management of chemical burns, where the risk of secondary exposure to hazardous substances poses a direct threat to the safety of rescue teams. Despite the urgency of treatment, proper donning of personal protective equipment is essential [87,88]. Swift and gentle removal of the victim’s clothing, while preventing further tissue damage, constitutes a critical component of early care [19,22,28,34,76]. These measures not only establish the groundwork for subsequent professional interventions but also help reduce long-term morbidity and may facilitate the necessary window for definitive burn treatment [87,88].

### 4.2. Prioritizing ABC Emergencies Under Resource-Constrained Conditions

Under conditions of limited resources—such as battlefield environments or mass-casualty incidents—triage must prioritize the management of ABC emergencies that pose an immediate threat to life [89]. This approach is consistent with fundamental trauma care principles and aligns with the “platinum ten minutes” and “golden hour” concepts, which emphasize the critical importance of rapid intervention to prevent avoidable mortality [90,91]. As a specific category of trauma, burn injuries are frequently complicated by fatal inhalation insults, necessitating timely identification and decisive management [92,93]. In this study, we summarize a concise set of clinical indicators and complementary diagnostic procedures for evaluating inhalation injury [18,23,29,75]. However, the scarcity of emergency care resources can create ambiguity in clinical judgment. Several studies have therefore quantified the risk and severity of inhalation injury, providing pragmatic assessment tools based on operationally accessible clinical indicators for use in resource-limited settings [94]. These tools allow responders to implement stratified treatment strategies in a targeted and efficient manner. Given the limited availability of oxygen in many prehospital settings, patients with moderate-to-severe inhalation injury should be prioritized for high-flow oxygen therapy at 10–15 L/min [29,75]. If adequate oxygenation cannot be maintained, advanced airway interventions—such as insertion of a nasopharyngeal airway or emergency cricothyroidotomy even invasive ventilatory support must be initiated without delay [29,75]. This step, although lifesaving, poses substantial technical challenges for primary healthcare providers and underscores the need for dedicated training in advanced airway management [95].

Circulatory resuscitation constitutes another critical component of early burn care, requiring a structured and context-appropriate treatment plan. In resource-limited environments, oral rehydration may be suitable for patients with burns covering <20% TBSA [19,25,32,41]. In contrast, patients with extensive burns (≥20% or ≥40% TBSA) should receive intravenous fluid resuscitation [18,19,22,23,25,28,41]. When intravenous access cannot be established promptly, intraosseous infusion provides a reliable alternative for fluid administration [19]. Regarding the volume of fluid resuscitation, the modified Brooke and Parkland formulas continue to serve as international standards in specialized burn centers [96]. However, our findings indicate the ten-fold formula suits the initial prehospital phase, especially for non-specialist frontline responders and existing studies confirm comparable accuracy to the two formulas mentioned above [25]. The formula’s simplicity—providing a consistent hourly fluid rate independent of weight variations—facilitates rapid calculation, minimizes dosing errors, and has demonstrated effectiveness in early resuscitation, supporting its broader dissemination [82]. In conclusion, prioritizing the assessment and management of life-threatening ABC conditions enables emergency personnel to convert the narrow window of critical intervention into meaningful survival gains. This strategy reduces the risk of secondary complications and lowers overall mortality among severely burned patients [97,98].

### 4.3. The Effectiveness of Burn Emergency Care Depends on Context-Adaptive, Resource-Based Interventions and Timely Transfer

While ABC management is focused on “saving lives,” subsequent interventions aim to “prolong life” and stabilize the patient for definitive treatment [24]. This phase requires context-adaptive implementation based on available resources rather than attempting comprehensive, hospital-level care in the field. Our evidence synthesis identifies several key domains for ongoing management—including hypothermia prevention, analgesia, wound care, infection control, and essential surgical measures—but emphasizes that successful practice depends on timely, situation-specific adjustments.

Burn patients, particularly those with extensive injuries (burns ≥ 20%) or concurrent seawater immersion, are highly susceptible to hypothermia due to loss of the skin barrier and evaporative heat loss—factors strongly associated with increased mortality [19,99,100]. In naval or maritime combat environments where seawater immersion is common and medical resources may be scarce, integrating continuous temperature monitoring with insulation or active rewarming strategies becomes essential [101]. These measures must be executed using whatever materials or equipment such as bedding and survival blankets are immediately available. Pain management for burn patients—including thresholds for intervention, modality selection, and stepwise analgesic escalation—generally follows established analgesic principles. The major challenge in resource-limited settings is the limited availability of potent opioid analgesics such as morphine [102]. During drug shortages, substituting agents such as ibuprofen or employing non-pharmacological interventions (e.g., psychological support, distraction, positioning) can offer meaningful relief from pain and anxiety when opioids are unavailable [103].

Current evidence generally supports the early application of cold therapy (ideally initiated within 10 min and no later than 3 h post-injury) using running water for approximately 20 min as a primary intervention for burns involving limited TBSA (often defined as <20%), with the understanding that the duration and feasibility of this approach should be adjusted based on available water resources and other operational constraints at the scene, followed by appropriate wound protection [19,25,26,33]. However, the choice of dressing for initial wound coverage remains a contentious issue and is heavily influenced by local resource availability. Determining the optimal dressing for burns of varying depths is a complex clinical decision, and no unified consensus currently exists. In resource-constrained settings, silver sulfadiazine (SSD) remains widely used—particularly for deep burns and exposure therapy—due to its affordability and broad antimicrobial properties [18,21,38,77]. Emerging evidence, however, suggests that certain novel dressings such as silver-containing hydrofiber dressings, nanocrystalline silver dressings, amniotic membranes, and other biological dressings may provide superior infection control and promote faster wound healing compared with SSD [104,105]. Additional high-quality studies are required to validate these findings and inform definitive recommendations. In practice, dressing choices can be pre-planned or upgraded based on operational needs and logistical feasibility. Surgical interventions represent the most technically demanding component of early burn care and are profoundly constrained by environmental sterility and the availability of skilled personnel. Procedures such as escharotomy for circumferential burns require experienced burn or trauma specialists capable of balancing sterility, hemostasis, and patient stability under austere conditions [106]. For patients with extensive burns, the highest priority is rapid transfer to a specialized burn center for definitive care, provided that the patient’s immediate safety can be ensured. Effective coordination of transportation and optimal allocation of limited resources serve as critical elements in preventing further clinical deterioration and improving overall prognosis [38,41].

In summary, this discussion reinforces the proposed framework for early management of burn patients in resource-limited settings. The core intervention—immediate cessation of the burn process combined with prompt assessment and monitoring—emerges as an unequivocal, non-negotiable priority across all contexts. At the first tier, life-saving measures such as airway management, respiratory support, and circulatory resuscitation are broadly applicable and must be prioritized under any resource condition. The second tier includes context-dependent interventions—hypothermia prevention, pain management, wound protection, surgical decompression, and infection control—whose implementation should be guided by available infrastructure and personnel expertise rather than pursued universally in the field. Meanwhile, transfer preparations, as well as nursing care and documentation, support the continuity of care and safe transitions. Across these levels, the evidence demonstrates strong consensus on core and first-tier interventions, but reveals significant limitations regarding second-tier practices, where high-quality studies are comparatively scarce—particularly comparing wound dressings, opioid-sparing pain strategies, and field feasibility of escharotomy. Clinically, these findings reinforce the need for pragmatic, resource-tiered protocols that enable primary healthcare providers and first responders to deliver life-sustaining care within realistic operational constraints.

## 5. Conclusions

This study provides a comprehensive synthesis of the best evidence for early management of burn injuries, with particular emphasis on the distinctive challenges encountered in resource-limited environments, such as battlefield environments or primary care facilities. By consolidating recommendations across 13 key domains and organizing them into immediate, first-aid, secondary-aid, and full-process care phases, this review bridges existing evidence with real-world operational constraints. The resulting synthesis offers structured, evidence-informed guidance tailored for primary healthcare providers and emergency responders. Implementing these practices may optimize the early management of adult burn patients, preserve critical time for definitive specialized care, and ultimately reduce long-term morbidity. In addition, this work establishes a foundational framework that can support the development of standardized clinical decision-making protocols for early burn care across diverse, resource-constrained settings. This synthesis is limited by the fact that the majority of included guidelines and primary studies originate from high-income settings, leaving their direct applicability to resource-constrained or austere environments largely untested. Future work should examine in greater depth the implications of implementing, validating in real-world settings, and locally adapting the proposed recommendations, thereby facilitating their effective translation across diverse operational contexts.

## Figures and Tables

**Table 1:** Quality evaluation results of guidelines (*n* = 10).

Included Literature	Standardized Score for Each Field (%)						≥60%	<30%	Quality
	Scope Purpose	Participants	Rigor	Clarity	Applicability	Independence			
Cartotto, R., 2024 [16]	96.30	61.11	55.56	87.04	59.72	8.33	3	1	B
Cartotto, R., 2023 [17]	96.30	66.67	60.42	87.04	66.67	8.33	5	1	B
Yoshino, Y., 2020 [18]	79.63	81.48	79.17	100.00	51.39	88.89	5	0	B
Romanowski, K.S., 2020 [12]	74.07	44.44	61.11	92.59	52.78	94.44	4	0	B
BBA, 2019 [19]	87.04	62.96	55.56	100.00	27.78	80.56	4	1	B
BBA, 2018 [20]	61.11	35.19	29.17	77.78	13.89	19.44	2	3	C
ISBI, 2018 [21]	83.33	98.15	53.47	88.89	68.06	52.78	4	0	B
EBA, 2017 [22]	85.19	81.48	44.44	81.48	75.00	50.00	4	0	B
ISBI, 2016 [23]	96.30	96.30	67.36	87.04	81.94	44.44	5	0	B
Velde, 2007 [24]	85.19	61.11	79.17	100	37.50	77.78	5	0	B

**Table 2:** Quality evaluation results of included systematic reviews (*n* = 24).

Included Literature	Q1	Q2	Q3	Q4	Q5	Q6	Q7	Q8	Q9	Q10	Q11
Hsiao, K.H., 2024 [51]	Y	Y	Y	Y	Y	Y	Y	Y	Y	Y	Y
Sarda, N.N., 2024 [52]	N	N	U	U	N	N	U	U	U	Y	N
Hsiao, K.H., 2024 [53]	Y	Y	Y	Y	Y	Y	Y	Y	Y	Y	N
Wenjie, L., et al., 2023 [54]	Y	Y	Y	Y	Y	Y	Y	Y	Y	Y	Y
Hafizurrachman, 2023 [55]	N	Y	Y	Y	Y	Y	U	U	N	Y	N
Abazari, M., 2022 [56]	N	N	N	U	U	N	N	U	U	Y	Y
Knappskog, K., 2022 [57]	Y	Y	Y	Y	Y	Y	Y	Y	U	Y	Y
Miroshnychenko, A., 2021 [58]	Y	Y	Y	Y	Y	Y	Y	Y	Y	Y	Y
Ziegler, B., 2020 [59]	Y	Y	Y	N	Y	U	Y	Y	U	Y	Y
Harshman Jamie, 2019 [60]	Y	Y	Y	Y	Y	Y	Y	Y	U	Y	Y
Jaspers, M.E.H., 2019 [61]	Y	Y	Y	Y	Y	Y	Y	Y	N	Y	Y
Reid, A., 2019 [63]	N	Y	Y	Y	Y	Y	Y	Y	U	Y	Y
Kwa, K.A.A., 2019 [62]	N	U	U	Y	N	N	N	U	U	Y	N
Morgan Michael, 2018 [69]	Y	Y	Y	Y	Y	Y	Y	Y	Y	Y	Y
Kao Yuan, 2018 [65]	U	U	Y	N	U	N	U	U	N	Y	Y
Scheffler, M., 2018 [66]	Y	Y	Y	Y	Y	Y	Y	Y	Y	Y	Y
Deutsch, C.J., 2018 [67]	Y	Y	Y	Y	Y	Y	Y	Y	Y	Y	Y
Nímia, H.H., 2018 [64]	N	Y	Y	Y	N	N	U	U	N	Y	Y
Yang Chao, 2018 [68]	N	Y	Y	Y	Y	U	Y	Y	Y	Y	Y
Ramos, G., 2017 [70]	Y	Y	Y	Y	Y	N	Y	Y	N	Y	Y
Goodwin, N.S., 2016 [71]	Y	Y	Y	Y	N	Y	Y	Y	U	Y	N
Wasiak, J., 2013 [72]	Y	Y	Y	Y	Y	Y	Y	Y	Y	Y	N
Aziz, Z., 2012 [73]	Y	Y	U	Y	Y	Y	Y	Y	Y	Y	Y
Avni, T., 2010 [74]	Y	Y	Y	Y	Y	U	Y	Y	N	Y	Y

Note: Y: Yes; N: No; U: Unclear; Q1: Was the evidence-based question posed clear and unambiguous? Q2: Were the inclusion criteria for the literature appropriate? Q3: Was the search strategy used appropriate? Q4: Were the sources of the research papers appropriate? Q5: Were the quality criteria of the literature appropriate? Q6: Was the quality assessment done independently by ≥2 researchers? Q7: Were measures used to minimize error when extracting data? Q8: Were the methods used for the synthesized/combined study accurate? Q9: Was there an assessment of possible publication bias? Q10: Were there any recommendations for policy and practice supported by reported data? Q11: Were there any recommendations made for further research directions?

**Table 3:** Summary of best evidence for early management of adult burn injury patients.

Category	Content of Evidence	Evidence Level	Recommended Level
Cessation of the Burn Process	1. Essential early actions include ensuring scene safety, extinguishing active fire sources, and preventing cross-contamination from chemicals, corrosive agents, or biological hazards [19]. The SAFE protocol (Shout, Assess, Free from danger, and Evaluate) may be applied to guide these steps [19].	5b	B
	2. The initial step is to remove the injured person from the combustion source or hazardous environment [21,25,26]. First responders must prioritize their own safety and that of bystanders at all times.	5b	A
	3. Clothing should be carefully cut away rather than pulled off. Any material adherent to the burn wound should be left in place and cooled as a composite layer to avoid additional tissue damage [19,22,28,34,76].	4c	B
Early Assessment and Monitoring	4. A systematic approach should be used to assess burn patients, with immediate attention given to life-threatening conditions [23,25]. Initial evaluation should follow the ABCDEF approach [23,28,34]. Secondary assessment includes obtaining a SAMPLE history, documenting the mechanism of injury [50], and performing a full body examination [34].	5b	A
	5. The risk and severity of inhalation injury should be assessed by considering whether the injury occurred in an enclosed space, exposure duration, the presence of open flames or explosions, and the potential involvement of accelerants, chemical agents, or toxic gases [29,75].	1a	A
	6. Standardized methods for estimating total body surface area (TBSA) include the rule of nines, the Lund–Browder chart, the palm method, and digital/computer-assisted tools [18,28,34]. The Lund-Browder chart is regarded as the most accurate method for determining TBSA, particularly in children [19,22,33].	5a	B
	7. Clinical symptom-based classification is recommended to assess the depth of burns [18].	4a	A
	8. Burn depth assessment may also be supported by adjunctive imaging or diagnostic technologies, including laser Doppler imaging (LDI) and video microscopy [18,22,26,61].	1c	B
	9. The Artz criteria—or modified systems such as the Moylan criteria—provide a structured framework for evaluating burn severity [18,22,26,61].	5a	B
	10. Telemedicine is advised for the initial assessment of severely burned patients [33].	5b	B
	11. Dynamic assessment of burn patients should include laboratory and physiologic monitoring, including hemoglobin/hematocrit, urea and creatinine, electrolytes, urinalysis, arterial blood gas analysis, and electrocardiography [34].	5b	B
Airway Management	12. Key indicators of respiratory injury include a history of exposure in an enclosed space; contact with high-temperature vapors, liquids, or explosion events; altered mental status; soot deposition in the oral cavity; facial or chest burns; singed nasal hair; dysphagia; hoarseness; carbonaceous sputum; wheezing; and dyspnea [18,23,29,75].	1a	A
	13. Chest radiography, computed tomography, and point-of-care ultrasound serve as important adjunctive tools for evaluating suspected airway burns [18,75].	3a	B
	14. Inhalation injuries are classified into three grades: mild injury confined to the upper airway; moderate injury involving the larynx and trachea; and severe injury extending to the lower airways, including the bronchi and pulmonary parenchyma [75].	5b	B
	15. In patients with obvious upper airway obstruction or impending asphyxia, immediate manual airway maneuvers should be undertaken to maintain patency and prevent respiratory arrest. If these are ineffective, advanced airway interventions—such as insertion of a nasopharyngeal airway or emergency cricothyroidotomy—may be required [25].	1a	A
	16. Endotracheal intubation or tracheotomy is recommended for patients with moderate to severe inhalation injury, especially when deep burns to the face or neck are present [18,19,25,33,75].	3b	A
Respiratory Support	17. Oxygen therapy is recommended for patients with mild to moderate inhalation injury, with high-flow oxygen (10–15 L/min) administered as clinically indicated. For patients with moderate to severe inhalation injury who fail to respond to high-concentration or high-flow oxygen, invasive ventilatory support should be initiated without delay [29,75].	5b	A
	18. Treatment for carbon monoxide or cyanide poisoning should be considered only in symptomatic patients with severe exposure [41]. Management includes high-flow oxygen therapy and administration of appropriate antidotal agents when indicated [19,33].	5b	A
Circulatory Resuscitation	19. Intravenous fluid resuscitation is the standard of care for patients with extensive burns [53]. Whenever possible, fluids should be administered through two large-caliber peripheral intravenous lines placed in unburned tissue [34]. If peripheral access is not feasible, intraosseous access is an appropriate alternative [19].	1a	A
	20. For patients with burns involving <20% TBSA, oral rehydration may be used for initial shock resuscitation under severe conditions [19,25,32,41]. If oral intake is ineffective or contraindicated, intravenous fluid therapy should be initiated [32].	5b	A
	21. In resource-limited settings, intravenous fluid resuscitation may need to be prioritized for patients with a higher probability of survival, such as those with <40% TBSA burn [18,19,22,23,25,28,41].	5b	A
	22. The “ten-fold” fluid replacement formula—Fluid rate (%TBSA × 10 mL/h)—is recommended for prehospital management of adult patients with extensive burns when frontline medical personnel are not burn specialists [82].	3c	A
	23. Isotonic electrolyte solutions are the preferred initial fluids for burn resuscitation [18,25,33]. Normal saline (0.9%) is not recommended due to the risk of hyperchloremic acidosis [22].	1b	A
	24. Blood transfusion is generally unnecessary during routine burn resuscitation unless there is a concurrent traumatic injury causing significant hemorrhage [19].	2c	B
	25. Fluid replacement should be titrated according to physiologic endpoints, including urine output, blood pressure, and heart rate [18,34]. In patients with severe burns, an indwelling urinary catheter should be placed to allow hourly monitoring. Fluid rates should be adjusted to maintain urine output at 0.5–1.0 mL/kg/h, heart rate <100 beats/min, and systolic blood pressure >100 mmHg [25].	5b	A
	26. Use of clinical decision support systems may be considered to reduce excessive fluid administration and optimize resuscitation accuracy [16].	5b	B
Hypothermia Prevention	27. Patients with burns involving ≥20% TBSA, as well as those exposed to seawater immersion, are at heightened risk for hypothermia [21]. Maintaining core body temperature and minimizing heat loss are essential during the acute phase of burn management [25].	5b	B
	28. Active rewarming should be initiated when core temperature falls below 36 °C. Core temperature should be monitored using reliable sites such as the tympanic membrane or rectum [25].	1c	A
	29. Surface rewarming includes placing the patient in a warm environment, covering burn wounds appropriately, and using warm blankets or external medical warming devices. Internal rewarming measures may include administering intravenous fluids warmed to 37 °C and providing warmed, humidified inhaled gas [79].	5b	B
	30. During wound cooling, care must be taken to keep uninjured skin dry. After cooling is complete, patients should be wrapped in clean sheets or blankets during ongoing management and transport [76].	2c	A
Pain Management	31. Analgesia is essential throughout all stages of burn care [19]. When patients report the need for pain relief or demonstrate a pain score greater than 3, an appropriate analgesic regimen should be initiated promptly [45].	5b	A
	32. Routine pain assessments, performed multiple times each day across all phases of treatment, are critical to effective pain management [12,21].	1c	A
	33. Pain assessment should be patient-centered, employing validated tools that align with individual communication abilities and clinical context [12,21].	3d	A
	34. The Numerical Rating Scale (NRS), along with facial-expression scales, the Visual Analogue Scale (VAS), and patient-reported intensity ratings, are recommended for evaluating burn pain [45]. The Critical Care Pain Observation Tool (CPOT) is appropriate for patients unable to communicate verbally [80].	5b	A
	35. The minimal effective opioid dose should be used for analgesia. Opioids should be combined with non-opioid pharmacologic agents and integrated with non-pharmacologic pain-management strategies [12].	3c	A
	36. Topical opioids may provide effective pain relief for burn wounds. For procedural or manipulation-related pain, fentanyl citrate oromucosal tablets and intranasal fentanyl serve as effective, noninvasive alternatives to oral opioid formulations [12].	1a	B
	37. Adjunctive non-pharmacological interventions—including virtual reality distraction therapy, hypnotherapy, music therapy, and combined relaxation–distraction techniques—have demonstrated efficacy in alleviating pain and reducing anxiety during wound care procedures [12,21,33,66,80].	1a	B
	38. Providing patient and family education regarding burn management and pain control, in conjunction with analgesic therapy, significantly reduces anxiety and improves pain outcomes [12,45,80].	2a	B
	39. Non-pharmacological strategies are recommended as the first-line approach for managing agitation and anxiety [21].	3c	A
	40. When pharmacologic sedation is required, structured sedation protocols and validated sedation scales should be used to guide titration and ensure that sedative dosing remains at the minimal effective level [21,29].	2d	A
	41. Mild sedation is preferred, allowing patients to be easily arousable and capable of following simple commands. When possible, non-benzodiazepine agents are recommended as first-line sedatives [21].	5b	B
Wound Management	42. Immediate cooling is recommended for adult patients with burns involving <20% TBSA who do not exhibit signs of shock [25,33]. Cooling is contraindicated in patients with burns >20% TBSA due to the risk of hypothermia [25,26].	5b	B
	43. Among available cooling methods, running cool water is the most effective [28]. The recommended duration of cooling is 20 min [26,28].	3c	B
	44. When running water is unavailable, acceptable alternatives include immersing the wound in water, applying a wet compress using a cold towel (changed every 15 s), spraying cool water, or using cooling hydrogel dressings [19,26,76].	5b	B
	45. For burns on the limbs, rinsing under running water is preferred. For burns involving the head, face, trunk, or groin, cold compresses with a wet towel may be used as clinically appropriate [26].	5b	B
	46. Optimal water temperature for cooling should not exceed 20 °C, with approximately 12 °C offering the best therapeutic effect. Water below 8 °C should be avoided because it increases the risk of tissue necrosis [19,76].	1c	B
	47. Burn cooling should be initiated as early as possible—ideally within 10 min of injury—and may remain beneficial for up to three hours post-injury [19,26,76].	3c	B
	48. Cooling should be monitored during prehospital care [19,24]. Non-cooled areas must be kept warm and dry to prevent hypothermia, and cooling must be stopped if the patient’s core temperature falls below 35 °C [28,76].	2c	B
	49. After cooling, burn wounds should be covered with clean, low-adhesive, moist dressings as temporary protection [19,24,26,76].	4c	B
	50. In prehospital settings, plastic food wrap (PVC film) can be used as a temporary cover for burn wounds, avoiding tight wrapping [19,22,28]. It should not be used on facial burns [76]. Home remedies should be strictly avoided [76].	5b	B
	51. If evacuation is expected within 24 h, blister skin should be preserved during initial out-of-hospital care [24,25,26,76]. Blisters that are ruptured, thin-walled, or contaminated should be carefully excised [24,26,76].	5b	B
	52. Burn wounds should be thoroughly cleansed before the application of appropriate dressings [22,23].	1a	A
	53. Within the first 48 h, wounds may be washed with saline or filtered tap water [22].	1b	A
	54. After initial cleansing, antimicrobial solutions such as chlorhexidine or dilute acetic acid may be used to target common contaminating organisms [22].	5b	B
	55. When evacuation within 24 h is not feasible due to limited resources, topical silver sulfadiazine powder may be applied. If resources are sufficient but evacuation is still delayed, wounds should be disinfected and bandaged with local antiseptic agents [25].	5b	B
	56. Non-blistering epidermal burns should be managed with moisturizing creams and appropriate patient education [34].	5b	B
	57. For superficial partial-thickness (superficial second-degree) burns with intact blisters, an oily cream or paraffin gauze may be applied after cleansing [18,26].	1b	A
	58. For superficial partial-thickness burns with blister skin removed, biological dressings are recommended after cleansing. Dressings that combine exudate absorption with moisture retention may also be used, along with oily creams and gauze [18,26].	5b	B
	59. Antibacterial dressings are recommended for wounds at risk of colonization or infection [22].	3a	A
	60. Silver-based agents and their alternatives are widely used for both partial- and full-thickness burn wounds. Silver sulfadiazine is commonly used for deeper wounds and in resource-limited settings [18,21,38,77].	1b	A
Infection Prevention and Control	61. Prophylactic systemic antibiotics are not recommended in the management of acute burn injuries [21,22,23,25,33,70].	1a	A
	62. For contaminated burns, tetanus prophylaxis should be administered using tetanus toxoid (TT) or human tetanus immunoglobulin (TIG) [18,23]. TIG is particularly indicated for patients with deep burns or shrapnel injuries [25].	4a	A
	63. For mild infections of superficial second-degree burns, management should primarily emphasize local wound care. For moderate infections, timely local interventions—including debridement and removal of necrotic tissue—combined with systemic antibacterial therapy are recommended. In cases of severe infection, immediate local treatment, urgent debridement, and concurrent systemic antimicrobial administration are essential [27].	5b	B
Surgical Disposal	64. Escharotomy is indicated when circumferential eschar on an extremity threatens distal perfusion or deep tissue viability, and when eschar on the chest, abdomen, or neck restricts ventilation or impairs respiratory function [18,19,22,23,25].	3c	B
	65. Fasciotomy is primarily indicated for compartment syndrome, most commonly associated with high-voltage electrical injuries or deep thermal burns [18,19,22,23,25].	2d	B
	66. Patients with severe burns should receive prompt surgical intervention for life-threatening complications. When chest trauma is present, closed thoracic drainage may be required to stabilize respiratory function [18,19,22,23,25].	5b	B
Basic Nursing Care	67. Maintaining a clean environment and strict adherence to hand hygiene protocols are essential for preventing cross-infection in patients with burn wounds [18,19,22,23,25].	3c	A
	68. Patients with burns involving the head—particularly those with inhalation injuries—should be positioned semi-recumbent (30–45°) or fully seated with the neck extended, with repositioning every two hours to optimize airway patency and pulmonary function [23,75].	1a	A
	69. For patients with circumferential burns of the chest or abdomen, a semi-recumbent position is recommended. Circumferentially burned or swollen limbs should be elevated to reduce edema and support circulation [22,39].	5b	A
	70. Early nutritional support is essential during the initial recovery phase [21,23] and should ideally begin within 12 h after injury [33].	5b	B
	71. Oral or enteral nutrition is preferred to parenteral nutrition whenever feasible, given its physiological, immunological, and metabolic advantages [33].	2a	A
	72. Patients with burns involving <10% TBSA may be managed with a regular diet [39]. For those with extensive burns (≥20% TBSA) or concomitant injuries, nasogastric feeding is recommended to ensure adequate caloric and protein intake [21,23].	5b	B
	73. When addressing psychological distress after burn injury, emotional and psychosocial aspects related to both the burn and its treatment should be considered and integrated into patient care [21,80].	3c	A
Emergency Care Documentation	74. Comprehensive documentation of burn injuries and their management is recommended and should include clinical photography to support accurate assessment and longitudinal monitoring [22,50].	5b	B
	75. A standardized burn area chart should be used to document the extent and distribution of burn injuries [28].	5b	B
	76. Standardized pain assessments should be performed and recorded throughout all phases of care to ensure timely and appropriate analgesic management [12,80].	5b	B
Post-Arrival and Transfer Preparations	77. Burn patients should be transported to a specialized burn center within 24 h whenever possible to allow for comprehensive evaluation and initiation of definitive care [12,80].	5b	A

## Data Availability

No new data were created or analyzed in this study. Data sharing is not applicable to this article.

## Supplementary Materials

The following supporting information can be downloaded at: https://www.mdpi.com/article/10.3390/ebj7020034/s1. File S1: Literature retrieval process; File S2: General information of the included literature; File S3: Literature quality appraisal; File S4: ICC of included guidelines; File S5: Evidence quality assurance and peer validation.

## Abbreviations

The following abbreviations are used in this manuscript:

LMICs	Low- and middle-income countries
JBI	Joanna Briggs Institute
NICE	National Institute for Health and Care Excellence
RNAO	Registered Nurses Association of Ontario
AHRQ	Agency for Healthcare Research and Quality
CNKI	China Knowledge Network
CBM	China Biomedical Literature Database
ISBI	International Society for Burn Injury
ABA	American Burn Association
TBSA	Total body surface area
LDI	Laser Doppler imaging
NRS	Numerical Rating Scale
VAS	Visual Analogue Scale
CPOT	Critical Care Pain Observation Tool
SSD	Silver sulfadiazine
