# Supplementary material for: Bridging the Evidence–Practice Gap in Early Burn Injury Care: A Comprehensive Evidence Synthesis of Global Guidelines, Consensus, and Systematic Reviews for Resource-Limited Settings
Source: Eur Burn J. 2026 Jun 10;7(2):34. doi: 10.3390/ebj7020034 (PMC13298258; doi:10.3390/ebj7020034)

File S1:Literature retrieval process

1. CBM （n=231）

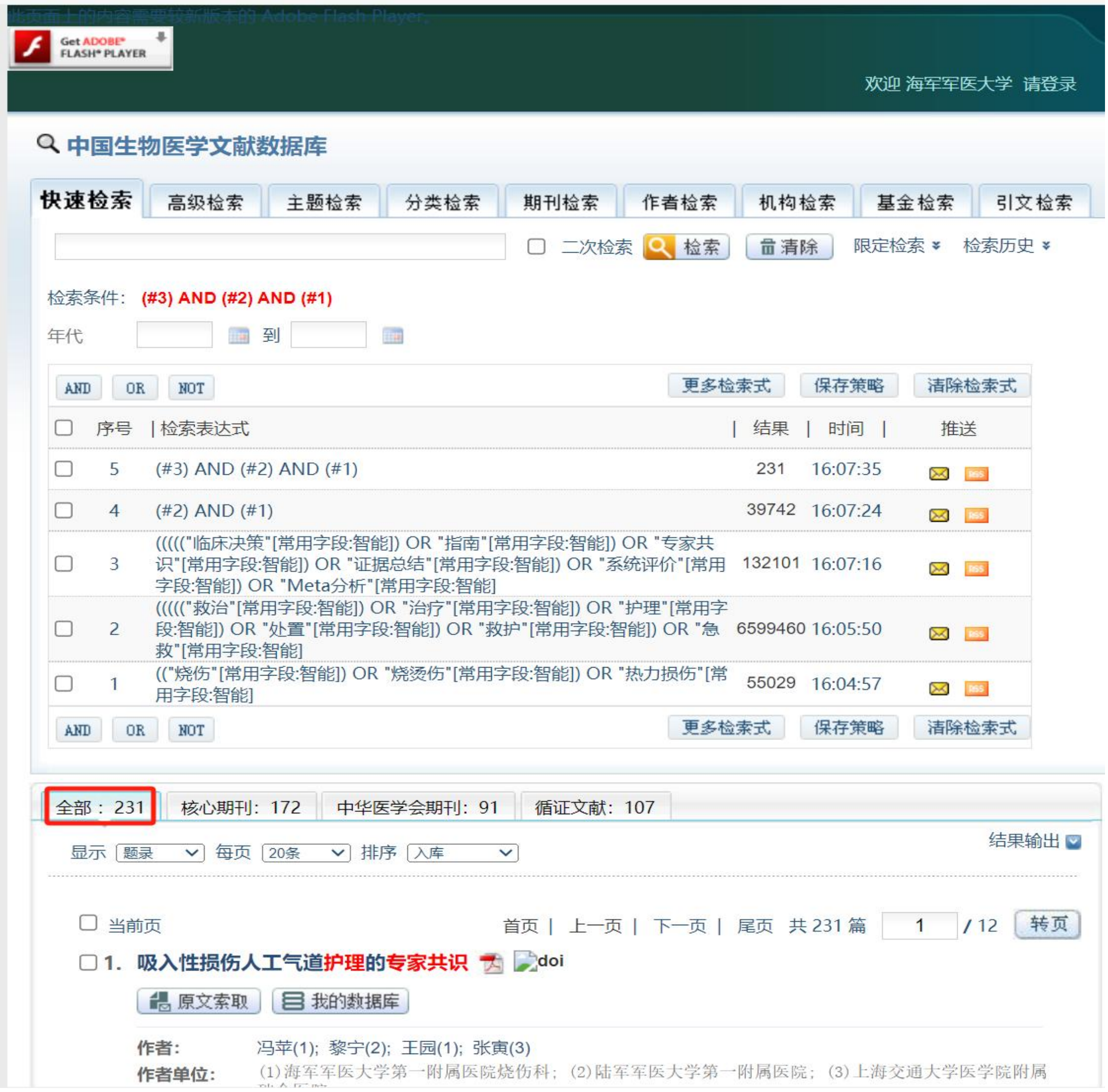

2. CNKI (n=62)

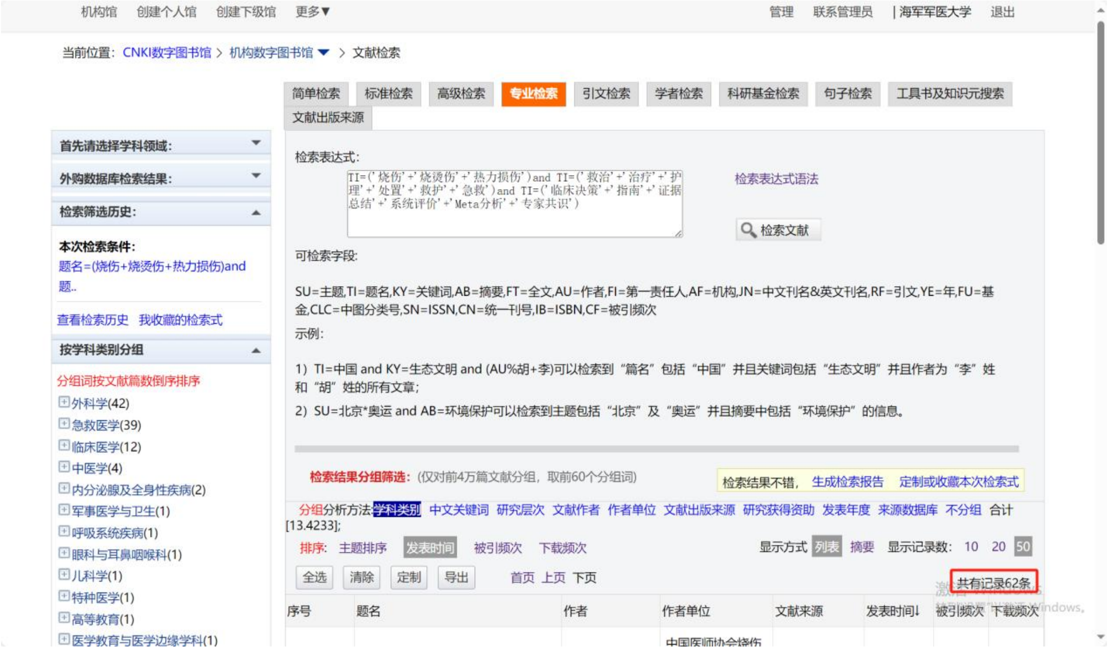

3. Yimaitong（70 items retrieved initially, 12 related； n=12）

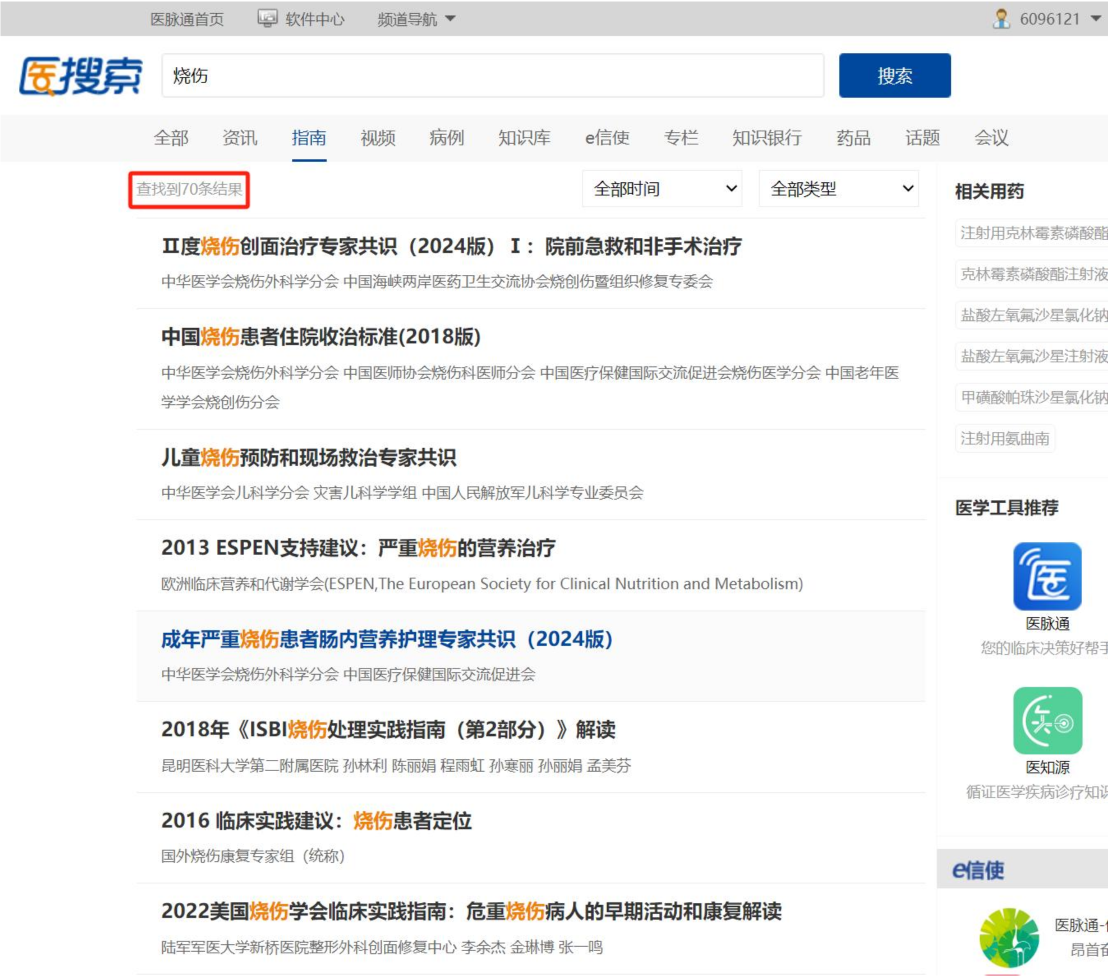

4. National Institute for Health and Care Excellence(NICE,https://www.nice.org.uk/)n=0

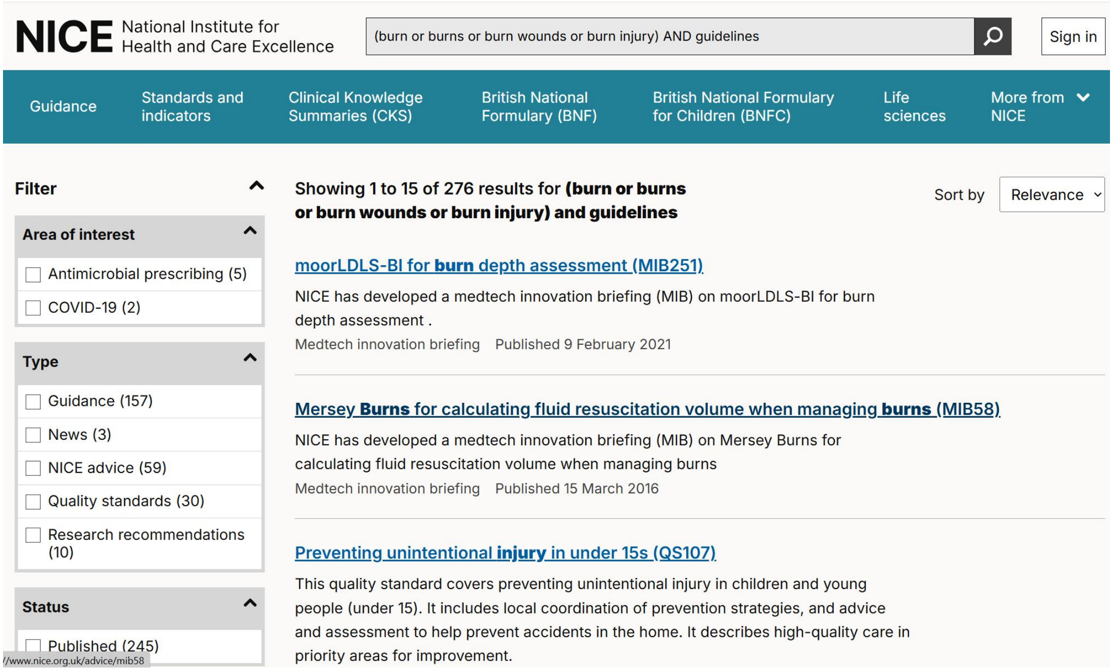

5. Registered Nurses Association of Ontario (RNAO,https://rnao.ca/) n=0

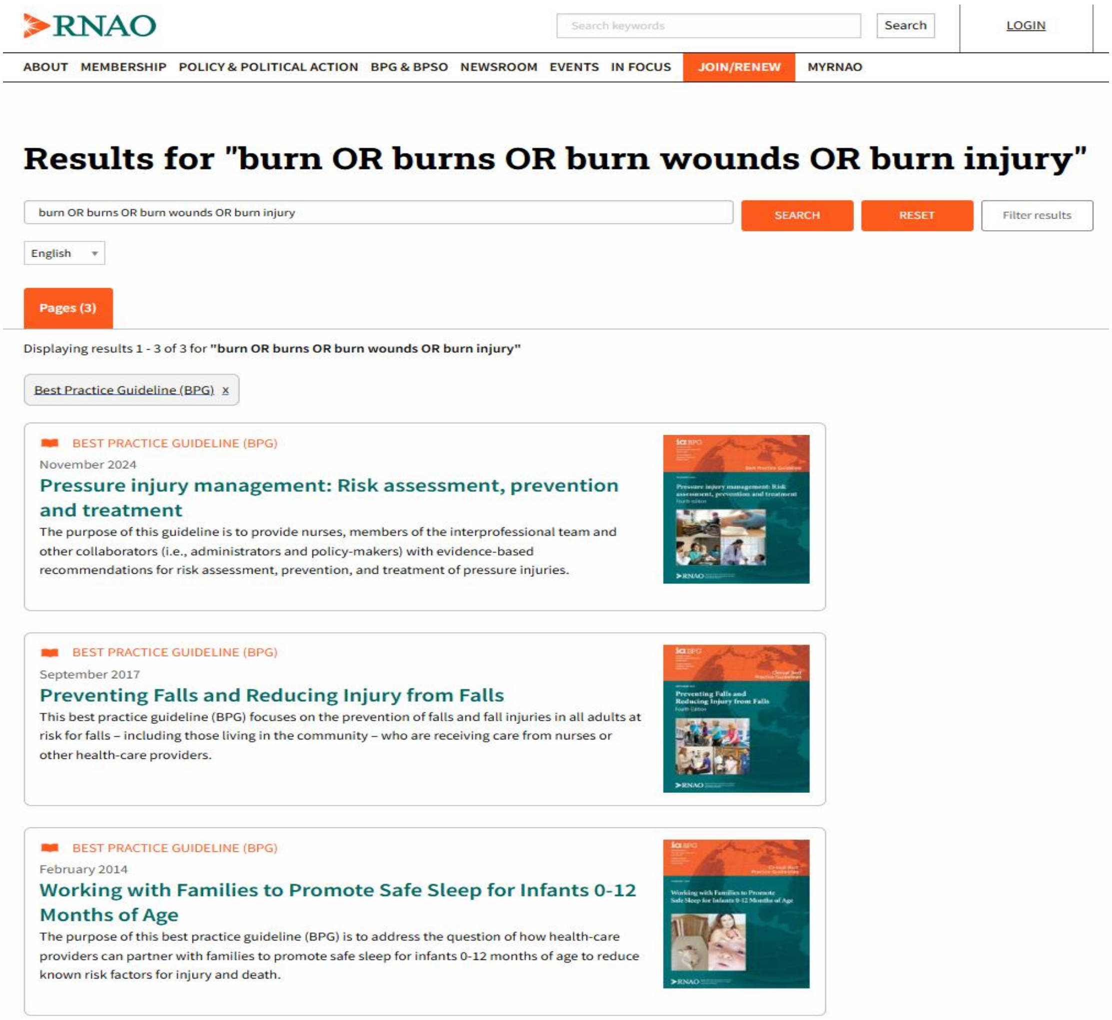

6. Agency for Healthcare Research and Quality (AHRQ,https://www.ahrq.gov/gam/index.html)n=0

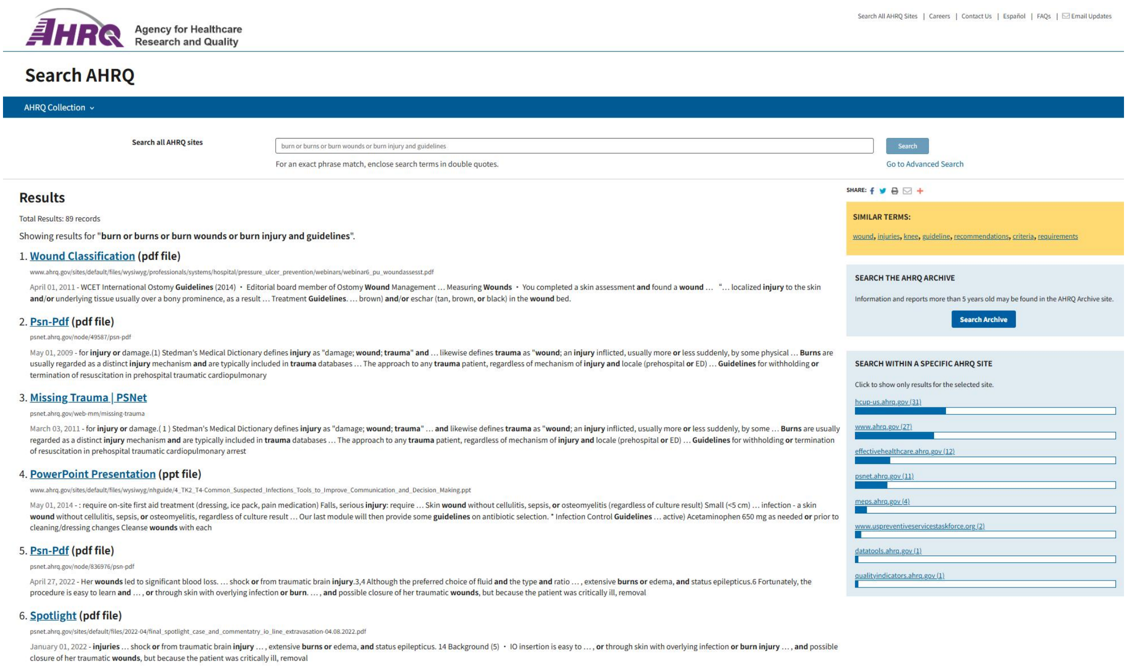

7. American Burn Association (ABA, https://www.ameriburn.org/)n=0

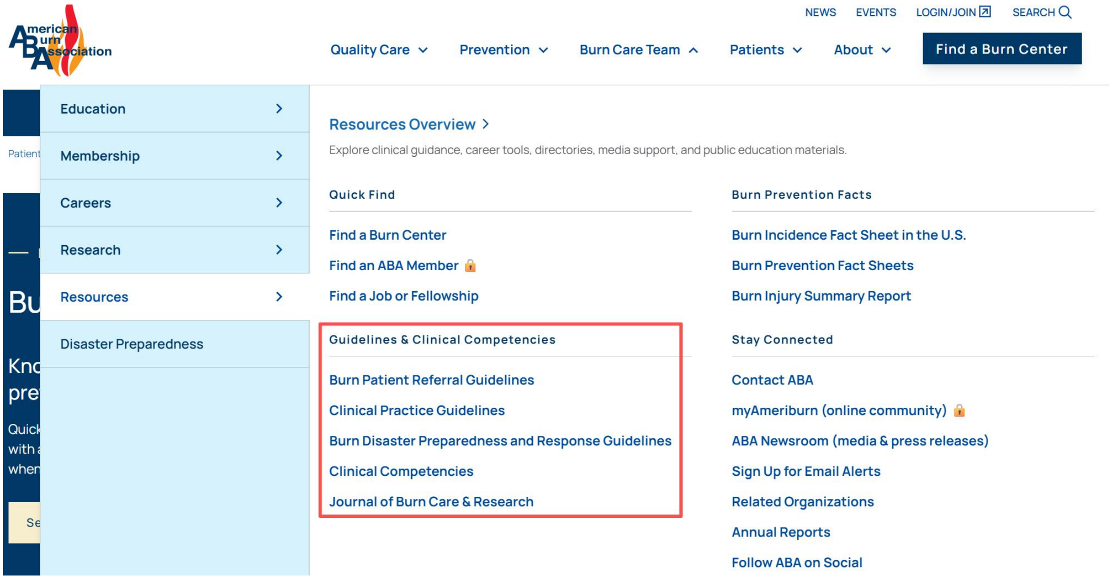

8. UpToDate (n=29)

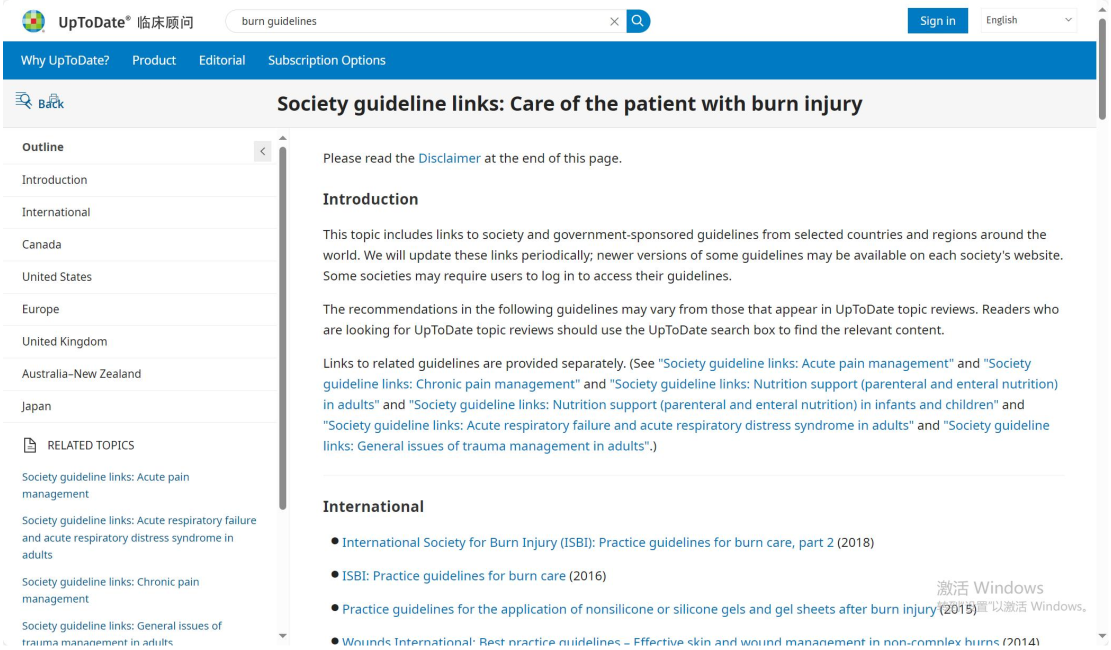

International

- International Society for Burn Injury (ISBI): Practice guidelines for burn care, part 2 (2018)
- ISBI: Practice guidelines for burn care (2016)
- Practice guidelines for the application of nonsilicone or silicone gels and gel sheets after burn injury (2015)
- Wounds International: Best practice guidelines – Effective skin and wound management in non-complex burns (2014)
- World Health Organization (WHO): A plan for burn prevention and care (2008)
- ISBI: Guidelines for dealing with disasters involving large numbers of extensive burns (2006)
- ~~International Commission for Mountain Emergency Medicine (ICAR) and International Mountaineering and Climbing Federation (UIAA): Official guidelines on lightning injuries – Prevention and on-site treatment in mountains and remote areas (2005)~~

United States

- American Burn Association (ABA): Clinical practice guidelines on burn shock resuscitation (2023)
- ABA: Clinical practice guideline – Early mobilization and rehabilitation of critically ill burn patients (2022)
- ABA: Position statement on home oxygen burn prevention (2022)
- ABA: Guidelines on the management of acute pain in the adult burn patient – A review of the literature, a compilation of expert opinion and next steps (2020)
- Western Trauma Association (WTA): Critical decisions in trauma – Preferred triage and initial management of the burned patient (2019)
- ABA: Guideline for burn care under austere conditions – Special care topics (2017)
- ABA: Guidelines for burn care under austere conditions – Special etiologies: Blast, radiation, and chemical injuries (2017)
- ABA: Guidelines for burn care under austere conditions – Introduction to burn disaster, airway and ventilator management, and fluid resuscitation (2016)
- ABA: Guidelines for burn care under austere conditions – Surgical and nonsurgical wound management (2016)
- American College of Surgeons (ACS): Statement on older adult burn prevention (2016)
- Wilderness Medical Society (WMS): Practice guidelines for basic wound management in the austere environment, update (2014)
- WMS: Practice guidelines for the prevention and treatment of lightning injuries, update (2014)
- ABA: Practice guidelines for prevention, diagnosis, and treatment of ventilator-associated pneumonia (VAP) in burn patients (2009)
- Wound Healing Society (WHS): Guidelines to aid healing of acute wounds by decreasing impediments of healing (2008)
- ABA/ACS: Guidelines for the operation of burn centers (2007)
- Practice guidelines for the management of electrical injuries (2006)

United Kingdom

- British Burn Association (BBA): Guidelines for pre-hospital care (first aid clinical practice guidelines)
- National Health System (NHS) Scotland: Care of Burns in Scotland (COBIS) – Clinical guidelines
- National Institute for Health and Care Excellence (NICE): Medical technologies guidance on moorLDI2-BI – A laser doppler blood flow imager for burn wound assessment (2011, updated 2017)
- NICE: Guideline on sunlight exposure – Risks and benefits (2016)
- NICE: Medical technologies guidance on the ReCell Spray-On Skin system for treating skin loss, scarring and depigmentation after burn injury (2014)
- Consensus on the prehospital approach to burns patient management (2004)

Europe

- European Wound Management Association (EWMA): Thermal burns – Practical recommendation for resource-limited setting
- European Resuscitation Council (ERC): Guidelines on cardiac arrest in special circumstances (2021)
- Eschar removal by bromelain based enzymatic debridement in burns: European consensus guidelines update (2020)
- European Burns Association (EBA): European practice guidelines for burn care – Minimum level of burn care provision in Europe (2017)
- Cyanide poisoning by fire smoke inhalation – A European expert consensus (2013)
- European Society for Clinical Nutrition and Metabolism (ESPEN): Endorsed recommendations – Nutritional therapy in major burns (2013)
- Pediatric cyanide poisoning by fire smoke inhalation – A European expert consensus (2013)
- European first aid guidelines (2007)

Australia–New Zealand

- New South Wales Agency for Clinical Innovation (NSW ACI): Burn transfer guidelines, 4th edition (amended 2022)
- ~~NSW ACI: Child life therapy – Burn patient management: Clinical guide (2020)~~
- NSW ACI: Clinical guidelines for burn patient management, 4th edition (2020)
- ~~NSW ACI: Donor site management for burn patients – A clinical guide (amended 2020)~~
- NSW ACI: Skin graft management for burn patients – A clinical guide (2020)
- NSW ACI: Clinical guidelines for escharotomy for burn patients, 2nd edition (amended 2019)
- NSW ACI: Clinical guidelines for minor burn management, 4th edition (amended 2019)
- NSW ACI: Burn physiotherapy and occupational therapy guidelines, 1st edition (amended 2017)
- ~~Department of Health, Government of South Australia (SA Health): Management of paediatric burns clinical guideline (2015)~~
- NSW ACI: Clinical practice guidelines for speech pathology burn patient management (2011)
- NSW ACI: Clinical practice guidelines on nutrition burn patient management (2011)
- NSW ACI: Clinical practice guidelines on social work (adults) burn patient management (2011)

9. International Society for Burn Injuries, ISBI（n=2）

ISBI

Contact the ISBI

Member Login

Home About ISBI News & Events Professional Resources Partnerships Awards, Grants & Scholarships Burn Center Assessment Program

Members 2025 ISBI Regional Conference World Burns Week

Home / Professional Resources / Education & Training / ISBI Practice Guidelines

English

Practice Guidelines (Part 1)

Practice Guidelines (Part 2)

Spanish

Practice Guidelines

Arabic

Practice Guidelines

10. JBI (initial search87 results, n=22 being relevant）

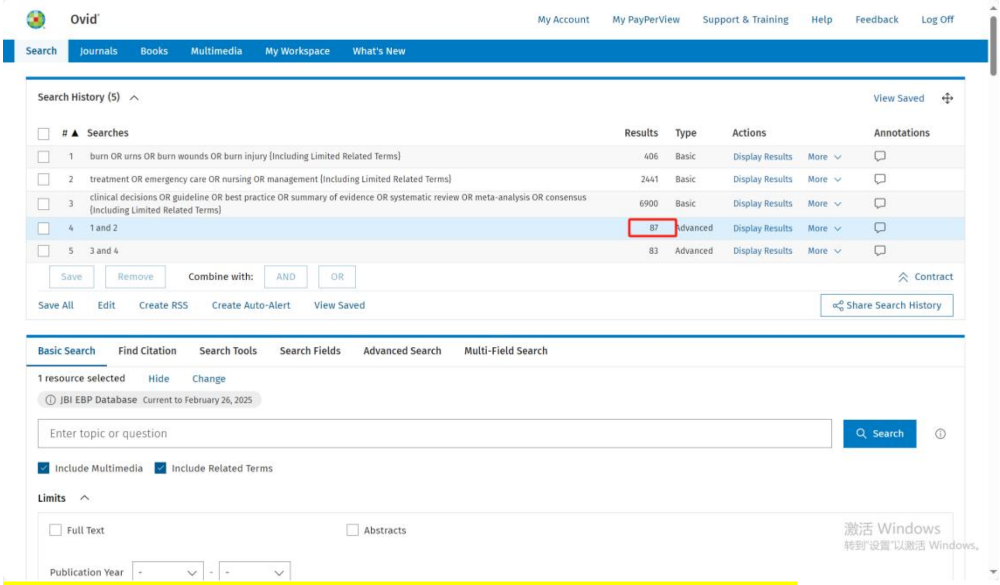

11.Cochrane library (initial search 139 results, n=12 being relevant）

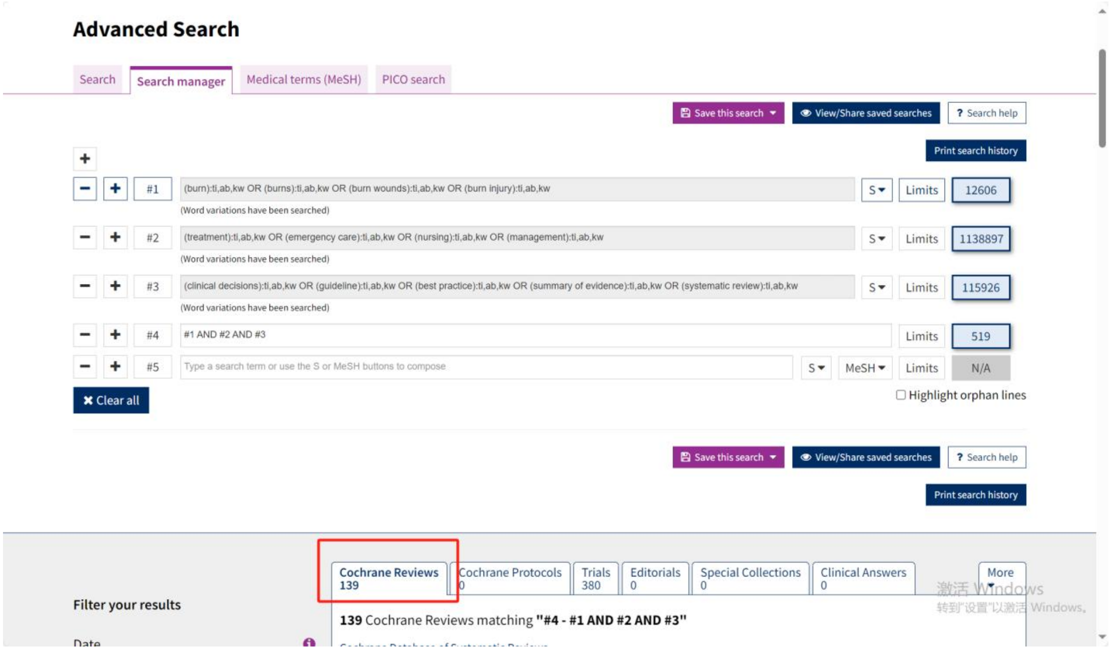

12.PUBMED (n=640)

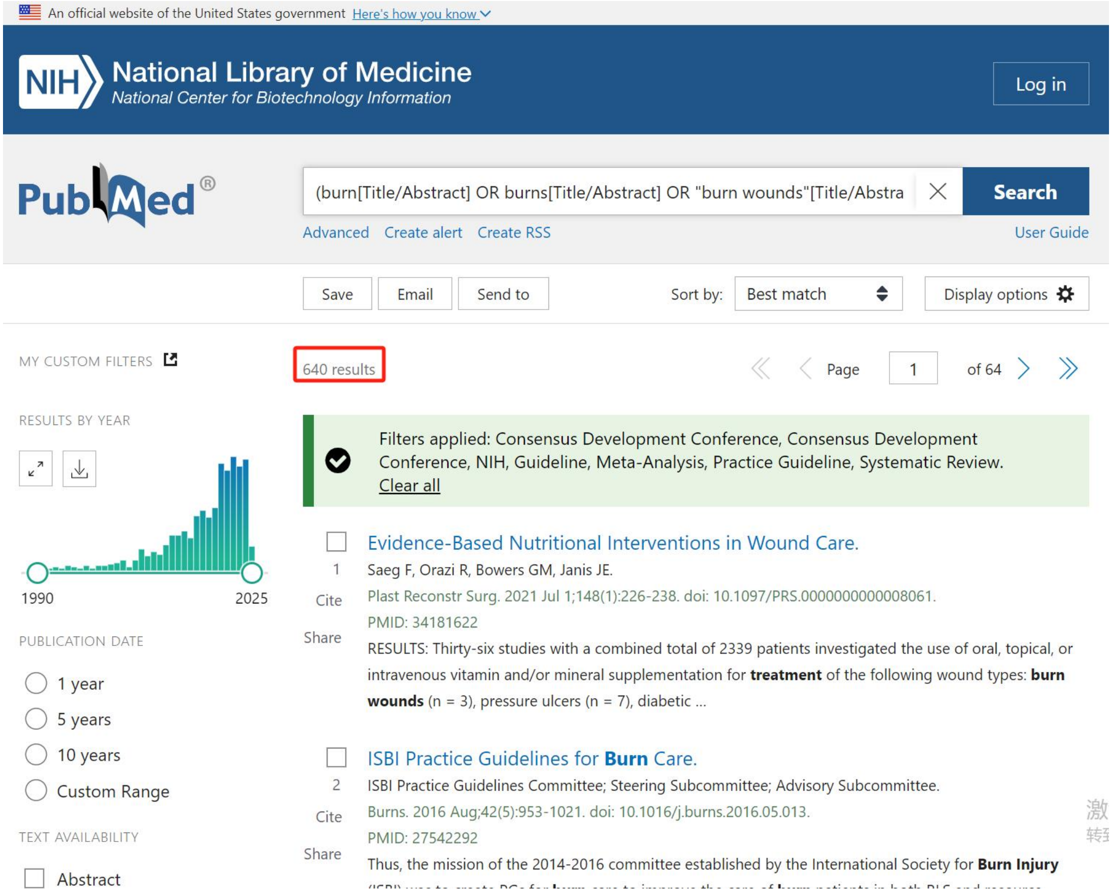

13.web of science (n=5003)

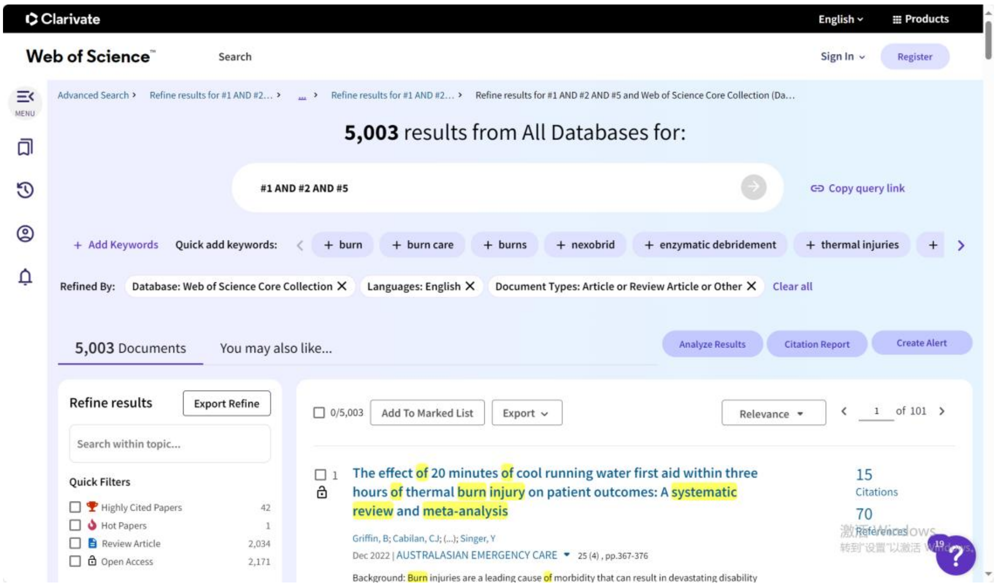

14.Embase (n=299)

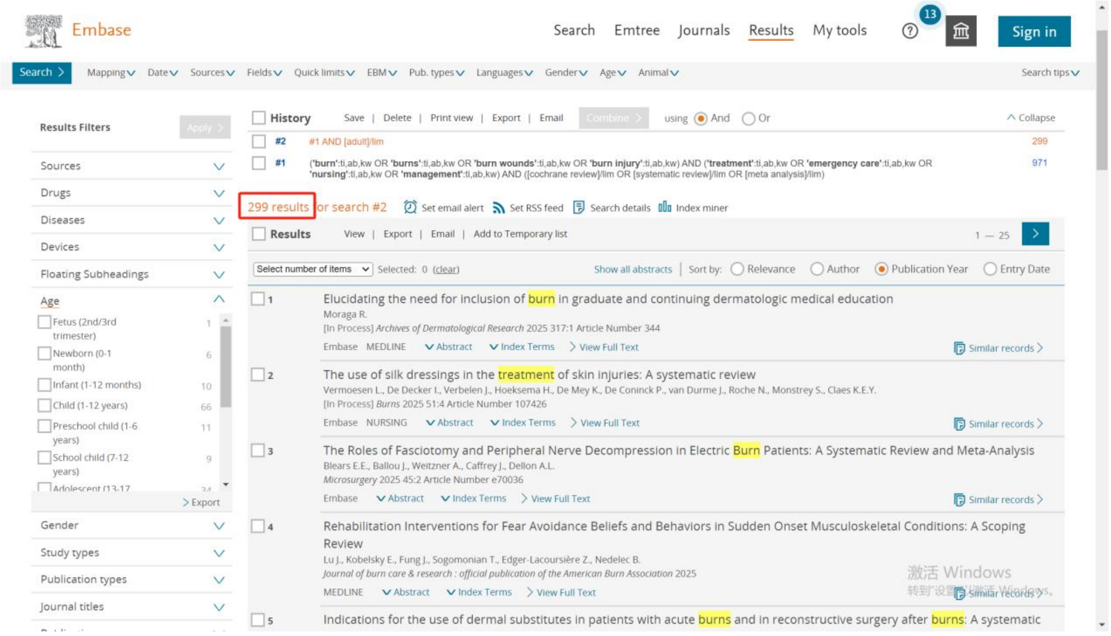

Supplement: Supplementary file 1 [file ebj-07-00034-s001.zip › File S1-Literature retrieval process.pdf]
