# Supplementary material for: Bridging the Evidence–Practice Gap in Early Burn Injury Care: A Comprehensive Evidence Synthesis of Global Guidelines, Consensus, and Systematic Reviews for Resource-Limited Settings
Source: Eur Burn J. 2026 Jun 10;7(2):34. doi: 10.3390/ebj7020034 (PMC13298258; doi:10.3390/ebj7020034)
Supplement: Supplementary file 1 [file ebj-07-00034-s001.zip › File S2-General information of the included literature.pdf]

File S2: General information of the included literature

	Author(year)	Study location	Literature source	Type	Literature subject
1	Cartotto R et al(2024)	America	Pubmed	Guideline	ABA Clinical Practice Guidelines on Burn Shock Resuscitation
2	Cartotto R et al(2023)	America	Pubmed	Guideline	Clinical Practice Guideline: Early Mobilization and Rehabilitation of Critically Ill Burn Patients
3	Yoshino Y et al(2020)	Japan	UpToDate	Guideline	Wound, pressure ulcer and burn guidelines: Guidelines for the management of burns
4	Romanowski KS et al(2020)	America	Pubmed	Guideline	ABA Guidelines on the Management of Acute Pain in the Adult Burn Patient: A Review of the Literature, a Compilation of Expert Opinion and Next Steps
5	BBA(2019)	UK	UpToDate	Guideline	Management of Burns in Pre - Hospital Trauma Care
6	BBA(2018)	UK		Guideline	BBA First Aid Clinical Practice Guidelinesnes
7	ISBI(2018)	America	ISBI	Guideline	ISBI Practice Guidelines for Burn Care, Part 2
8	EBA(2017)	Netherlands	UpToDate	Guideline	European practice quidelines for burn care - Minimum level of burn care provision in Europe
9	ISBI(2016)	America	ISBI	Guideline	ISBI Practice Guidelines for Burn Care
10	Velde(2007)	Belgium	Pubmed	Guideline	European first aid guidelines
11	SMEA(2025)	China	CNKI	Expert consensus	Expert consensus on early treatment of burns in ship environment
12	CBA(2024)	China	CNKI	Expert consensus	Expert consensus on the treatment of second-degree burn wounds Ⅰ: pre-hospital first aid and non-surgical treatment
13	CBA(2024)	China	CNKI	Expert consensus	Expert consensus on the treatment of second-degree burn wounds Ⅱ : surgical treatment and infection prevention and treatment
14	NSW ACI(2024)	Austrilia	UpToDate	Expert consensus	Clinical guidelines for minor burn management, 4th edition
15	Feng P et al(2023)	China	CNKI	Expert consensus	Expert consensus on care of artificial airway for inhalation injury
16	Zhu F et al(2022)	China	CBM	Expert consensus	National expert consensus on proneposition therapy in adult burn patients
17	Wang Y et al(2021)	China	CNKI	Expert consensus	National expert consensus on the clinical application of eschar dermabrasion in bum wounds
18	BTB-CGS(2020)	China	CBM	Expert consensus	National experts consensus on prevention and treatment of burn shock
19	Legrand M et al(2020)	America	Pubmed	Expert consensus	Management of severe thermal burns in the acute phase in adults and children
20	NSW ACI(2019)	Austrilia	UpToDate	Expert consensus	Clinical guidelines for burn patient management, 4th edition
21	NSW ACI(2019)	Austrilia	UpToDate	Expert consensus	Clinical quidelines for escharotomy for burn patients
22	BTB-CGS(2018)	China	CBM	Expert consensus	National experts consensus on tracheotomy and intubation for burn patients
23	Driscoll IR et al(2018)	America	Pubmed	Expert consensus	Burn Casualty Care in the Deployed Setting
24	Cancio LC et al(2017)	America	Pubmed	Expert consensus	Guidelines for Burn Care Under Austere Conditions: Surgical and Nonsurgical Wound Management
25	Young AW et	America	Pubmed	Expert	Guideline for Burn Care Under Austere Conditions:

	Author(year)	Study location	Literature source	Type	Literature subject
	al(2017)			consensus	Special Care Topics
26	Cancio LCet al(2017)	America	Pubmed	Expert consensus	Guidelines for Burn Care Under Austere Conditions: Special Etiologies: Blast, Radiation, and Chemical Injuries
27	Kearns RD et al(2016)	America	Pubmed	Expert consensus	Guidelines for Burn Care Under Austere Conditions: Introduction to Burn Disaster, Airway and Ventilator Management, and Fluid Resuscitation
28	Yasti AC et al(2015)	Turkey	Pubmed	Expert consensus	Guideline and treatment algorithm for burn injuries
29	Atiyeh B et al(2014)	UK	UpToDate	Expert consensus	International Best Practice Guidelines: Effective skin and wound management of non complex burns
30	Luo GX et al(2014)	China	pubmed	Expert consensus	Guideline for diagnosis, prophylaxis and treatment of invasive fungal infection post burn injury in China 2013.
31	Editorial Board of Chinese Journal of Burns(2013)	China	CNKI	Expert consensus	Guidelines for pain management in adult burns
32	CMDA(2012)	China	CNKI	Expert consensus	Diagnostic criteria and treatment guidelines for burn infections
33	NSW ACI(2011)	Austrilia	UpToDate	Expert consensus	Clinical practice guidelines on nutrition burn patient management
34	Alsbjörn B et al(2007)	Denmark	pubmed	Expert consensus	Guidelines for the management of partial-thickness burns in a general hospital or community setting-recommendations of a European working party
35	Haberal M et al(2006)	Turkey	pubmed	Expert consensus	Guidelines for dealing with disasters involving large numbers of extensive burns
36	Allison K et al(2004)	UK	pubmed	Expert consensus	Consensus on the prehospital approach to burns patient management
37	Chen WY et al(2023)	China	CNKI	Evidence summary	Best Evidence Summary of Airway Management in Inhalation Injury
38	Liu GZ et al(2023)	China	CNKI	Evidence summary	Summary of the best evidence for emergency wound management in adult burn patients
39	Garcia G et al(2022)	America	Pubmed	Evidence summary	Topical Antimicrobial Agents for the Prevention of Burn-Wound Infection. What Do International Guidelines Recommend? A Systematic Review
40	Cheng YH et al(2020)	China	CNKI	Evidence summary	Summary of the best evidence for management of tracheotomy in burn patients with inhalation injury
41	Lu FC et al(2020)	China	CNKI	Evidence summary	Evidence summary for postoperative hypothermia rewarming in adults with severe burns
42	Chen C et al(2017)	China	CNKI	Evidence summary	Nursing and management of wound procedural pain in adult burn patients：evidence synthesis
43	Wasiak J et al(2009)	Australia	Pubmed	Evidence summary	Burns (minor thermal)
44	Hsiao KH et al(2024)	Switzerlan d	Pubmed	Systematic review	Adapted approaches to initial fluid management of patients with major burns in resource-limited settings: A systematic review
45	Sarda NN et al(2024)	India	Pubmed	Systematic review	Screening and Phasewise Management of Burn Injuries
46	Hsiao KH et	Switzerlan	Pubmed	Systematic	Oral/enteral fluid resuscitation in the initial

	Author(year)	Study location	Literature source	Type	Literature subject
	al(2024)	d		review	management of major burns: A systematic review and meta-analysis of human and animal studies
47	Li WJ et al(2023)	China	CNKI	Systematic review	Effects of different dressings in the treatment of burn wounds: a network meta-analysis
48	Hafizurrachman M et al(2023)	Indonesia	Pubmed	Systematic review	Review of Traditional First Aid for Burn Injuries in the 21st Century
49	Abazari M et al(2022)	Iran	Pubmed	Systematic review	A Systematic Review on Classification, Identification, and Healing Process of Burn Wound Healing
50	Knappskog K et al(2022)	Norway	Pubmed	Systematic review	Vasoactive and/or inotropic drugs in initial resuscitation of burn injuries: A systematic review
51	Miroshnychenko A et al(2021)	Canada	EMBASE	Systematic review	Comparison of early surgical intervention to delayed surgical intervention for treatment of thermal burns in adults: A systematic review and meta-analysis
52	Ziegler B et al(2020)	Germany	Pubmed	Systematic review	Evidence and Trends in Burn Wound Debridement: An Evidence Map
53	Harshman J et al(2019)	Canada	Pubmed	Systematic review	Emergency Care of the Burn Patient Before the Burn Center: A Systematic Review and Meta-analysis
54	Nímia HH et al(2019)	Brazil	Pubmed	Systematic review	Comparative study of Silver Sulfadiazine with other materials for healing and infection prevention in burns: A systematic review and meta-analysis
55	Jaspers MEH et al(2019)	Netherlands	Pubmed	Systematic review	A systematic review on the quality of measurement techniques for the assessment of burn wound depth or healing potential
56	Reid A et al(2019)	Australia	Pubmed	Systematic review	Inhalational injury and the larynx: A review
57	Kwa KAA et al(2019)	Netherlands	Pubmed	Systematic review	A systematic review on surgical and nonsurgical debridement techniques of burn wounds
58	Morgan Michael et al(2018)	Australia	Pubmed	Systematic review	Burn Pain: A Systematic and Critical Review of Epidemiology, Pathophysiology, and Treatment
59	Kao Yuan et al(2018)	Taiwan	Pubmed	Systematic review	Fluid Resuscitation in Patients With Severe Burns: A Meta-analysis of Randomized Controlled Trials
60	Scheffler M et al(2018)	Germany	Pubmed	Systematic review	Efficacy of non-pharmacological interventions for procedural pain relief in adults undergoing burn wound care: A systematic review and meta-analysis of randomized controlled trials
61	Deutsch CJ et al(2018)	UK	Pubmed	Systematic review	The diagnosis and management of inhalation injury: An evidence based approach
62	Yang C et al(2018)	China	Pubmed	Systematic review	Efficacy and feasibility of opioids for burn analgesia: An evidence-based qualitative review of randomized controlled trials
63	Ramos G et al(2017)	Argentina	Pubmed	Systematic review	Systemic antimicrobial prophylaxis in burn patients: systematic review
64	Goodwin NS et al(2016)	Australia	Pubmed	Systematic review	The efficacy of hydrogel dressings as a first aid measure for burn wound management in the pre-hospital setting: a systematic review of the literature
65	Wasiak J et al(2013)	Australia	Cochrane	Systematic review	Dressings for superficial and partial thickness burns
66	Aziz Z et al(2012)	Malaysia	Pubmed	Systematic	A systematic review of silver-containing dressings and

	Author(year)	Study location	Literature source	Type	Literature subject
				review	topical silver agents (used with dressings) for burn wounds
67	Avni T et al(2010)	Israel	Pubmed	Systematic review	Prophylactic antibiotics for burns patients: systematic review and meta-analysis

American Burn Association(ABA); British Burns Association(BBA); European Burns Association(EBA); Cross-Straits Medicine Exchange Association(SMEA);Chinese Burn Association(CBA);Burn and Trauma Branch of the Chinese Geriatrics Society(BTB-CGS); Agency for Clinical Innovation, New South Wales(NSW ACI);Chinese Medical Doctor Association(CMDA)
