# Supplementary material for: Bridging the Evidence–Practice Gap in Early Burn Injury Care: A Comprehensive Evidence Synthesis of Global Guidelines, Consensus, and Systematic Reviews for Resource-Limited Settings
Source: Eur Burn J. 2026 Jun 10;7(2):34. doi: 10.3390/ebj7020034 (PMC13298258; doi:10.3390/ebj7020034)
Supplement: Supplementary file 1 [file ebj-07-00034-s001.zip › File S4-ICC of included guidelines.pdf]

File S4: ICC of included guidelines (n =10)

Guidelines			ICC	P
**1**	Cartotto R,2024	American Burn Association Clinical Practice Guidelines on Burn Shock Resuscitation	0.949	＜0.001
**2**	Cartotto R,2023	Clinical Practice Guideline: Early Mobilization and Rehabilitation of Critically Ill Burn Patients	0.973	＜0.001
**3**	Yoshino Y，2020	Wound, pressure ulcer and burn guidelines-6: Guidelines for the management of burns, second edition	0.901	＜0.001
**4**	Romanowski KS，2020	American Burn Association Guidelines on the Management of Acute Pain in the Adult Burn Patient: A Review of the Literature, a Compilation of Expert Opinion and Next Steps	0.822	＜0.001
**5**	BBA，2019	Management of Burns in Pre - Hospital Trauma Care	0.942	＜0.001
**6**	British Burns Association,2018	British Burn Association (BBA):Guidelines for pre-hospital care first aid clinical practice guidelines	0.361	0.013
**7**	ISBI，2018	ISBI Practice Guidelines Committee.ISBI Practice Guidelines for Burn Care, Part 2	0.835	＜0.001
**8**	European Burns Association，2017	European Burns Association (EBA): European practice quidelines for burn care - Minimum level of burn care provision in Europe	0.802	＜0.001
**9**	ISBI，2016	ISBI Practice Guidelines for Burn Care	0.925	＜0.001
**10**	Velde，2007	European first aid guidelines	0.952	＜0.001
