# Supplementary material for: Bridging the Evidence–Practice Gap in Early Burn Injury Care: A Comprehensive Evidence Synthesis of Global Guidelines, Consensus, and Systematic Reviews for Resource-Limited Settings
Source: Eur Burn J. 2026 Jun 10;7(2):34. doi: 10.3390/ebj7020034 (PMC13298258; doi:10.3390/ebj7020034)
Supplement: Supplementary file 1 [file ebj-07-00034-s001.zip › File S5-Evidence quality assurance and peer validation.pdf]

**Table S1. Basic Information of the Expert Panel.**

| No. | Gender | Age (year) | Positional Title | Educational background | Years of Work Experience | Professional Field |
|---|---|---|---|---|---|---|
| 1 | Male | 48 | Associate Senior Physician | PhD | 25 | Burn Medicine，Acute and Chronic Wound Management |
| 2 | Female | 48 | Associate Senior Nurse | BD | 28 | Critical Care Nursing，Nursing Management |
| 3 | Female | 34 | Associate Senior Nurse | MD | 11 | Burn Specialty Nursing，Wound Management，Nursing Management |
| 4 | Female | 34 | Associate Professor | PhD | 10 | Trauma Emergency Care，Disaster Medicine and Emergency Rescue |
| 5 | Female | 32 | Supervisor Nurse | MD | 11 | Burn Specialty Nursing，Wound Management，Nursing Management |

## Expert Consultation on Optimal Evidence-Based Recommendations for Early Care of Adult Burns under Resource-Limited Conditions

This consultation form is based on the evidence synthesis method in evidence-based medicine, compiling evidence entries related to burn care under resource-limited conditions, particularly prior to specialized burn treatment.

Our research team had previously classified the evidence levels (Levels 1–5) of the original studies according to the Australian JBI Evidence-Based Practice Centre criteria, with lower numerical values indicating higher evidence strength. Based on four dimensions—feasibility, appropriateness, effectiveness, and clinical significance—the team comprehensively assessed the strength of evidence recommendations, categorizing them as Level A recommendations (strong recommendation) and Level B recommendations (weak recommendation).

We now invite you as an expert to re-evaluate the rationality of the strength recommendation predictions for each piece of evidence in Column 4 of Table 1, and fill in the review result (A or B) in Column 5. Please make a comprehensive judgment based on the feasibility, appropriateness, effectiveness, and clinical significance of the evidence items.

**Table S2. Expert Verification of Evidence Strength Recommendations.**

| Category | Content of evidence | Evidence Level | Recommeded level | Expert Verification results |
|---|---|---|---|---|
| Cessation of the Burn Process | 1. Essential early actions include ensuring scene safety, extinguishing active fire sources, and preventing cross-contamination from chemicals, corrosive agents, or biological hazards. The SAFE protocol (Shout, Assess, Free from danger, and Evaluate) may be applied to guide these steps. | 5b | B | B |
| | 2. The initial step is to remove the injured person from the combustion source or hazardous environment. First responders must prioritize their own safety and that of bystanders at all times. | 5b | A | A |
| | 3. Clothing should be carefully cut away rather than pulled off. Any material adherent to the burn wound should be left in place and cooled as a composite layer to avoid additional tissue damage. | 4c | A | B |
| Early Assessment and Monitoring | 4. A systematic approach should be used to assess burn patients, with immediate attention given to life-threatening conditions. Initial evaluation should follow the ABCDEF approach[23, 28, 34]. Secondary assessment includes obtaining a SAMPLE history, documenting the mechanism of injury[50], and performing a full body examination. | 5b | A | A |
| | 5. The risk and severity of inhalation injury should be assessed by considering whether the injury occurred in an enclosed space, exposure duration, the presence of open flames or explosions, and the potential involvement of accelerants, chemical agents, or toxic gases. | 1a | A | A |
| | 6. Standardized methods for estimating total body surface area (TBSA) include the rule of nines, the Lund–Browder chart, the palm method, and digital/computer-assisted tools. The Lund-Browder chart is regarded as the most accurate method for determining TBSA, particularly in children. | 5a | B | B |
| | 7. Clinical symptom-based classification is recommended to assess the depth of burns. | 4a | A | A |
| | 8. Burn depth assessment may also be supported by adjunctive imaging or diagnostic technologies, including laser Doppler imaging (LDI) and video microscopy. | 1c | B | B |
| | 9. The Artz criteria—or modified systems such as the Moylan criteria—provide a structured framework for evaluating burn severity. | 5a | B | B |
| | 10.Telemedicine is advised for the initial assessment of severely burned patients. | 5b | B | B |
| | 11. Dynamic assessment of burn patients should include laboratory and physiologic monitoring, including hemoglobin/hematocrit, urea and creatinine, electrolytes, urinalysis, arterial blood gas analysis, and electrocardiography. | 5b | B | B |
| Airway Management | 12. Key indicators of respiratory injury include a history of exposure in an enclosed spaces; contact with high-temperature vapors, liquids, or explosion events; altered mental status; soot deposition in the oral cavity; facial or chest burns; singed nasal hair; dysphagia; hoarseness; carbonaceous sputum; wheezing; and dyspnea. | 1a | A | A |
| | 13. Chest radiography, computed tomography, and point-of-care ultrasound serve as important adjunctive tools for evaluating suspected airway burns. | 3a | B | B |
| | 14. Inhalation injuries are classified into three grades: mild injury confined to the upper airway; moderate injury involving the larynx and | 5b | B | B |

| Category | Content of evidence | Evidence Level | Recommeded level | Expert Verification results |
| --- | --- | --- | --- | --- |
| | trachea; and severe injury extending to the lower airways, including the bronchi and pulmonary parenchyma. | | | |
| | 15. In patients with obvious upper airway obstruction or impending asphyxia, immediate manual airway maneuvers should be undertaken to maintain patency and prevent respiratory arrest. If these are ineffective, advanced airway interventions—such as insertion of a nasopharyngeal airway or emergency cricothyroidotomy—may be required. | 1a | A | A |
| | 16. Endotracheal intubation or tracheotomy is recommended for patients with moderate to severe inhalation injury, especially when deep burns to the face or neck are present. | 3b | A | A |
| Respiratory Support | 17. Oxygen therapy is recommended for patients with mild to moderate inhalation injury, with high-flow oxygen (10–15 L/min) administered as clinically indicated. For patients with moderate to severe inhalation injury who fail to respond to high-concentration or high-flow oxygen, invasive ventilatory support should be initiated without delay. | 5b | B | A |
| | 18. Treatment for carbon monoxide or cyanide poisoning should be considered only in symptomatic patients with severe exposure. Management includes high-flow oxygen therapy and administration of appropriate antidotal agents when indicated. | 5b | B | A |
| Circulatory Resuscitation | 19. Intravenous fluid resuscitation is the standard of care for patients with extensive burns. Whenever possible, fluids should be administered through two large-caliber peripheral intravenous lines placed in unburned tissue. If peripheral access is not feasible, intraosseous access is an appropriate alternative. | 1a | A | A |
| | 20. For patients with burns involving <20% TBSA, oral rehydration may be used for initial shock resuscitation under severe conditions. If oral intake is ineffective or contraindicated, intravenous fluid therapy should be initiated. | 5b | A | A |
| | 21. In resource-limited settings, intravenous fluid resuscitation may need to be prioritized for patients with a higher probability of survival, such as those with <40% TBSA burn. | 5b | B | A |
| | 22. The "ten-fold" fluid replacement formula—Fluid rate (%TBSA × 10 mL/h)—is recommended for prehospital management of adult patients with extensive burns when frontline medical personnel are not burn specialists. | 3c | A | A |
| | 23. Isotonic electrolyte solutions are the preferred initial fluids for burn resuscitation. Normal saline (0.9%) is not recommended due to the risk of hyperchloremic acidosis. | 1b | A | A |
| | 24. Blood transfusion is generally unnecessary during routine burn resuscitation unless there is a concurrent traumatic injury causing significant hemorrhage. | 2c | A | B |
| | 25. Fluid replacement should be titrated according to physiologic endpoints, including urine output, blood pressure, and heart rate. In patients with severe burns, an indwelling urinary catheter should be placed to allow hourly monitoring. Fluid rates should be adjusted to maintain urine output at 0.5–1.0 mL/kg/h, heart rate <100 beats/min, and systolic blood pressure >100 mmHg. | 5b | B | A |
| | 26. Use of clinical decision support systems may be considered to reduce excessive fluid administration and optimize resuscitation accuracy. | 5b | B | B |
| Hypothermia | 27. Patients with burns involving ≥20% TBSA, as well as those exposed | 5b | B | B |

| Category | Content of evidence | Evidence Level | Recommeded level | Expert Verification results |
|---|---|---|---|---|
| Prevention | to seawater immersion, are at heightened risk for hypothermia. Maintaining core body temperature and minimizing heat loss is essential during the acute phase of burn management. | | | |
| | 28. Active rewarming should be initiated when core temperature falls below 36°C. Core temperature should be monitored using reliable sites such as the tympanic membrane or rectum. | 1c | A | A |
| | 29. Surface rewarming includes placing the patient in a warm environment, covering burn wounds appropriately, and using warm blankets or external medical warming devices. Internal rewarming measures may include administering intravenous fluids warmed to 37°C and providing warmed, humidified inhaled gas. | 5b | B | B |
| | 30. During wound cooling, care must be taken to keep uninjured skin dry. After cooling is complete, patients should be wrapped in clean sheets or blankets during ongoing management and transport. | 2c | A | A |
| Pain Management | 31. Analgesia is essential throughout all stages of burn care[19]. When patients report the need for pain relief or demonstrate a pain score greater than 3, an appropriate analgesic regimen should be initiated promptly. | 5b | B | A |
| | 32. Routine pain assessments, performed multiple times each day across all phases of treatment, are critical to effective pain management. | 1c | A | A |
| | 33. Pain assessment should be patient-centered, employing validated tools that align with individual communication abilities and clinical context. | 3d | A | A |
| | 34. The Numerical Rating Scale (NRS), along with facial-expression scales, the Visual Analogue Scale (VAS), and patient-reported intensity ratings, are recommended for evaluating burn pain[45]. The Critical Care Pain Observation Tool (CPOT) is appropriate for patients unable to communicate verbally. | 5b | A | A |
| | 35. The minimal effective opioid dose should be used for analgesia. Opioids should be combined with non-opioid pharmacologic agents and integrated with non-pharmacologic pain-management strategies. | 3c | A | A |
| | 36. Topical opioids may provide effective pain relief for burn wounds. For procedural or manipulation-related pain, fentanyl citrate oromucosal tablets and intranasal fentanyl serve as effective, noninvasive alternatives to oral opioid formulations. | 1a | B | B |
| | 37. Adjunctive non-pharmacological interventions—including virtual reality distraction therapy, hypnotherapy, music therapy, and combined relaxation–distraction techniques—have demonstrated efficacy in alleviating pain and reducing anxiety during wound care procedures. | 1a | B | B |
| | 38. Providing patient and family education regarding burn management and pain control, in conjunction with analgesic therapy, significantly reduces anxiety and improves pain outcomes. | 2a | A | B |
| | 39. Non-pharmacological strategies are recommended as the first-line approach for managing agitation and anxiety. | 3c | A | A |
| | 40. When pharmacologic sedation is required, structured sedation protocols and validated sedation scales should be used to guide titration and ensure that sedative dosing remains at the minimal effective level. | 2d | A | A |
| | 41. Mild sedation is preferred, allowing patients to be easily arousable and capable of following simple commands. When possible, non-benzodiazepine agents are recommended as first-line sedatives. | 5b | B | B |

| Category | Content of evidence | Evidence Level | Recommeded level | Expert Verification results |
|---|---|---|---|---|
| Wound Management | 42. Immediate cooling is recommended for adult patients with burns involving <20% TBSA who do not exhibit signs of shock. Cooling is contraindicated in patients with burns >20% TBSA due to the risk of hypothermia. | 5b | A | B |
| | 43. Among available cooling methods, running cool water is the most effective. The recommended duration of cooling is 20 minutes. | 3c | A | B |
| | 44. When running water is unavailable, acceptable alternatives include immersing the wound in water, applying a wet compress using a cold towel (changed every 15 seconds), spraying cool water, or using cooling hydrogel dressings. | 5b | B | B |
| | 45. For burns on the limbs, rinsing under running water is preferred. For burns involving the head, face, trunk, or groin, cold compresses with a wet towel may be used as clinically appropriate. | 5b | A | B |
| | 46. Optimal water temperature for cooling should not exceed 20°C, with approximately 12°C offering the best therapeutic effect. Water below 8°C should be avoided because it increases the risk of tissue necrosis. | 1c | B | B |
| | 47. Burn cooling should be initiated as early as possible—ideally within 10 minutes of injury—and may remain beneficial for up to three hours post-injury. | 3c | A | B |
| | 48. Cooling should be monitored during prehospital care. Non-cooled areas must be kept warm and dry to prevent hypothermia, and cooling must be stopped if the patient's core temperature falls below 35°C. | 2c | A | B |
| | 49. After cooling, burn wounds should be covered with clean, low-adhesive, moist dressings as temporary protection. | 4c | B | B |
| | 50. In prehospital settings, plastic food wrap (PVC film) can be used as a temporary cover for burn wounds, avoiding tight wrapping. It should not be used on facial burns[76]. Home remedies should be strictly avoided. | 5b | B | B |
| | 51. If evacuation is expected within 24 hours, blister skin should be preserved during initial out-of-hospital care. Blisters that are ruptured, thin-walled, or contaminated should be carefully excised. | 5b | B | B |
| | 52. Burn wounds should be thoroughly cleansed before the application of appropriate dressings. | 1a | A | A |
| | 53. Within the first 48 hours, wounds may be washed with saline or filtered tap water . | 1b | A | A |
| | 54. After initial cleansing, antimicrobial solutions such as chlorhexidine or dilute acetic acid may be used to target common contaminating organisms. | 5b | B | B |
| | 55. When evacuation within 24 hours is not feasible due to limited resources, topical silver sulfadiazine powder may be applied. If resources are sufficient but evacuation is still delayed, wounds should be disinfected and bandaged with local antiseptic agents. | 5b | A | B |
| | 56. Non-blistering epidermal burns should be managed with moisturizing creams and appropriate patient education. | 5b | B | B |
| | 57. For superficial partial-thickness (superficial second-degree) burns with intact blisters, an oily cream or paraffin gauze may be applied after cleansing. | 1b | A | A |
| | 58. For superficial partial-thickness burns with blister skin removed, biological dressings are recommended after cleansing. Dressings that combine exudate absorption with moisture retention may also be used, | 5b | B | B |

| Category | Content of evidence | Evidence Level | Recommeded level | Expert Verification results |
|---|---|---|---|---|
| | along with oily creams and gauze. | | | |
| | 59. Antibacterial dressings are recommended for wounds at risk of colonization or infection. | 3a | A | A |
| | 60. Silver-based agents and their alternatives are widely used for both partial- and full-thickness burn wounds. Silver sulfadiazine is commonly used for deeper wounds and in resource-limited settings. | 1b | A | A |
| Infection Prevention and Control | 61. Prophylactic systemic antibiotics are not recommended in the management of acute burn injuries . | 1a | A | A |
| | 62. For contaminated burns, tetanus prophylaxis should be administered using tetanus toxoid (TT) or human tetanus immunoglobulin (TIG). TIG is particularly indicated for patients with deep burns or shrapnel injuries. | 4a | A | A |
| | 63. For mild infections of superficial second-degree burns, management should primarily emphasize local wound care. For moderate infections, timely local interventions—including debridement and removal of necrotic tissue—combined with systemic antibacterial therapy are recommended. In cases of severe infection, immediate local treatment, urgent debridement, and concurrent systemic antimicrobial administration are essential. | 5b | B | B |
| Surgical Disposal | 64. Escharotomy is indicated when circumferential eschar on an extremity threatens distal perfusion or deep tissue viability, and when eschar on the chest, abdomen, or neck restricts ventilation or impairs respiratory function. | 3c | B | B |
| | 65. Fasciotomy is primarily indicated for compartment syndrome, most commonly associated with high-voltage electrical injuries or deep thermal burns. | 2d | A | B |
| | 66. Patients with severe burns should receive prompt surgical intervention for life-threatening complications. When chest trauma is present, closed thoracic drainage may be required to stabilize respiratory function. | 5b | B | B |
| Basic Nursing Care | 67. Maintaining a clean environment and strict adherence to hand hygiene protocols are essential for preventing cross-infection in patients with burn wounds. | 3c | A | A |
| | 68. Patients with burns involving the head—particularly those with inhalation injuries—should be positioned semi-recumbent (30°–45°) or fully seated with the neck extended, with repositioning every two hours to optimize airway patency and pulmonary function. | 1a | A | A |
| | 69. For patients with circumferential burns of the chest or abdomen, a semi-recumbent position is recommended. Circumferentially burned or swollen limbs should be elevated to reduce edema and support circulation. | 5b | A | A |
| | 70. Early nutritional support is essential during the initial recovery phase and should ideally begin within 12 hours after injury. | 5b | B | B |
| | 71. Oral or enteral nutrition is preferred to parenteral nutrition whenever feasible, given its physiological, immunological, and metabolic advantages. | 2a | A | A |
| | 72. Patients with burns involving <10% TBSA may be managed with a | 5b | B | B |

| Category | Content of evidence | Evidence Level | Recommeded level | Expert Verification results |
| --- | --- | --- | --- | --- |
| | regular diet. For those with extensive burns (≥20% TBSA) or concomitant injuries, nasogastric feeding is recommended to ensure adequate caloric and protein intake. | | | |
| | 73. When addressing psychological distress after burn injury, emotional and psychosocial aspects related to both the burn and its treatment should be considered and integrated into patient care. | 3c | A | A |
| Emergency Care Documentation | 74. Comprehensive documentation of burn injuries and their management is recommended and should include clinical photography to support accurate assessment and longitudinal monitoring. | 5b | B | B |
| | 75. A standardized burn area chart should be used to document the extent and distribution of burn injuries. | 5b | B | B |
| | 76. Standardized pain assessments should be performed and recorded throughout all phases of care to ensure timely and appropriate analgesic management. | 5b | B | B |
| Post-Arrival and Transfer Preparations | 77. Burn patients should be transported to a specialized burn center within 24 hours whenever possible to allow for comprehensive evaluation and initiation of definitive care. | 5b | B | A |
